# Microstructure and composition dependence of mechanical characteristics of nanoimprinted AlCoCrFeNi high-entropy alloys

**DOI:** 10.1038/s41598-021-93272-y

**Published:** 2021-07-01

**Authors:** Dinh-Quan Doan, Te-Hua Fang, Tao-Hsing Chen

**Affiliations:** 1 Department of Mechanical Engineering, National Kaohsiung University of Science and Technology, Kaohsiung, 807 Taiwan; 2 Faculty of Mechanical Engineering, Hung Yen University of Technology and Education, Khoai Chau District, Hung Yen Province Vietnam

**Keywords:** Mechanical engineering, Nanoscale materials, Nanoparticles, Structural properties

## Abstract

Molecular dynamics is applied to explore the deformation mechanism and crystal structure development of the AlCoCrFeNi high-entropy alloys under nanoimprinting. The influences of crystal structure, alloy composition, grain size, and twin boundary distance on the mechanical properties are carefully analyzed. The imprinting load indicates that the highest loading force is in ascending order with polycrystalline, nano-twinned (NT) polycrystalline, and monocrystalline. The change in alloy composition suggests that the imprinting force increases as the Al content in the alloy increases. The reverse Hall–Petch relation found for the polycrystalline structure, while the Hall–Petch and reverse Hall–Petch relations are discovered in the NT-polycrystalline, which is due to the interactions between the dislocations and grain/twin boundaries (GBs/TBs). The deformation behavior shows that shear strain and local stress are concentrated not only around the punch but also on GBs and adjacent to GBs. The slide and twist of the GBs play a major in controlling the deformation mechanism of polycrystalline structure. The twin boundary migrations are detected during the nanoimprinting of the NT-polycrystalline. Furthermore, the elastic recovery of material is insensitive to changes in alloy composition and grain size, and the formability of the pattern is higher with a decrease in TB distance.

## Introduction

Over the past decades, a variety of materials have been widely developed with the continuous efforts of scientists and engineers to expand their applications. The composition of traditional alloys includes one major element and other trace elements. The addition of trace elements has the aim of effectively improving the properties of alloys^1^. Likewise, increasing the number of main elements in an alloy produces a multi-principal element alloy with various characteristics. The production of multi-principal element alloys generates a new approach to the fabrication of many novel alloy materials^1,2^.

A novel designed layer of high-entropy alloys (HEAs) is gaining increasing attention due to their proprietary construction and excellent mechanical properties^3–6^. The HEAs contain five or more principal elements with a share of 5–35% each in molar ratios. These major elements deputize a new metallurgical field that focuses on the center rather than merely the edges and corners of the phase diagram^2,4,7^. Besides considering the different content of alloying elements in the new alloy design concept, it also clarifies the basic physics issues such as configurable entropy, phase selection, and energy free^8,9^. The formation of simple solid solution phase with multi principal elements in HEA is enhanced by highly mixed entropy coupled with the slow diffusion of the atoms^10,11^. These novel compositions provide expanded solid solution phases with single or matrix phase at the atomic level in the structures of face-centered cubic (FCC), body-centered cubic (BCC), and hexagonal close-packing (HCP), so the HEAs possess unique characteristics^12,13^. The detection of HEAs provides a novel alloy concept, from which a significant number of original alloys are formed, such as high-entropy ceramic, high-entropy bulk metallic glass^14–16^. The HEAs have shown unique characteristics that may not be present in other materials, which are precisely attractive for several specific environmental and engineering applications. For example, the HEAs possess extremely high hardness^17^, high thermal stability^18^, special thermophysical and magnetic properties^19^, and excellent anticorrosive properties^20^. The impressive characteristics of HEAs make them promising engineering material for utilization in a wide variety of applications such as fracture resistant material^7^, irradiation resistance material^21^, and tool material^22^.

Among the HEAs, AlCoCrFeNi HEA has attracted a large amount of attention due to its excellent mechanical properties^23–25^, and many studies have determined that the concentration of Al has a noticeable effect on the modifying phase structures. Li et al.^26^ studied Al_x_CoCrFeNi HEAs with x values from 1 to 3 in a molar ratio. The results indicated that Al promotes the formation of BCC structure, especially the absence of Cu in the alloy. The increase of x leads to deformity of the crystal lattice and the alloy reinforcement. Shi et al.^27^ investigated the effects of Al addition on the crystal structure, microstructure, and mechanical property of Al_x_CoCrFeNi HEAs. They showed that the microstructure changes from single solid-solution to multi-phases with increasing the Al content leading to the segregations of the elements, and the corrosion resistance of the surface passive film is reduced as the Al content in the alloy increases. Chao et al.^28^ revealed that the increased Al content in Al_x_CoCrFeNi HEAs results in a decrease in the microstructure stability of the coating and thus a higher degree of thermal softening under-treated isotherms at 1000 °C. Nevertheless, experimental observations for Al_x_CoCrFeNi HEAs are not all available, which lead to the evolution of other means such as molecular dynamics (MD) simulation.

MD simulation as an effective tool is broadly applied to handle the mechanical characteristics and microscopic structural evolution in materials^29–31^. Xie et al.^32^ showed the deposition resulting in the growth of HEA clusters, and coalescence phenomenon occurred with additional annealing between 300 and 1500 K during the annealing processes of AlCoCrCuFeNi HEA using MD simulation. Alhafez et al.^33^ explored the plastic response of CoCrFeMnNi HEA by means of nanoindentation tests through MD simulations, and their results indicated significant differences in identified plastic behavior. Furthermore, the nanoimprinting process has been one of the most attractive technologies for manufacturing nano-scale samples. The nanoimprinting can provide the simplest operation, high resolution, low cost, high throughput^34,35^. Due to the demand for super high resolution with economic efficiency and mass production capability in flexible electronics, semiconductor, and data storage industries, nanoimprinting is one of the most promising key technologies to fulfil the requirements^36–38^.

However, very little literature has focused on the microscopic behavior of Al_x_CoCrFeNi HEAs subject to nanoimprinting deformation. Besides, it is difficult to directly observe the microstructure development during the nanoimprinting of Al_x_CoCrFeNi HEAs by experimental method. To further explore the mechanical characteristics of Al_x_CoCrFeNi HEAs, MD simulation is used to probe the deformation behavior and microstructure development under the nanoimprinting process in the present work. The influences of different crystal structures of Al_x_CoCrFeNi HEA including monocrystalline, polycrystalline, and nano-twinned polycrystalline (NT-polycrystalline) on mechanical characteristics are compared in detail. In addition, the effects of alloy composition, grain size, and twin boundary (TB) distance on the mechanistic reaction in nanoimprinting also evaluate. The results show that the crystal structure, alloy composition, grain size, and TB distance have an apparent influence on microstructure development, imprinting force, and elastic recovery. Furthermore, the grain and twin boundaries (GBs/TBs) play important roles in nanoimprinting.

## Results

### Influence of crystal structure

**Figure 1:**
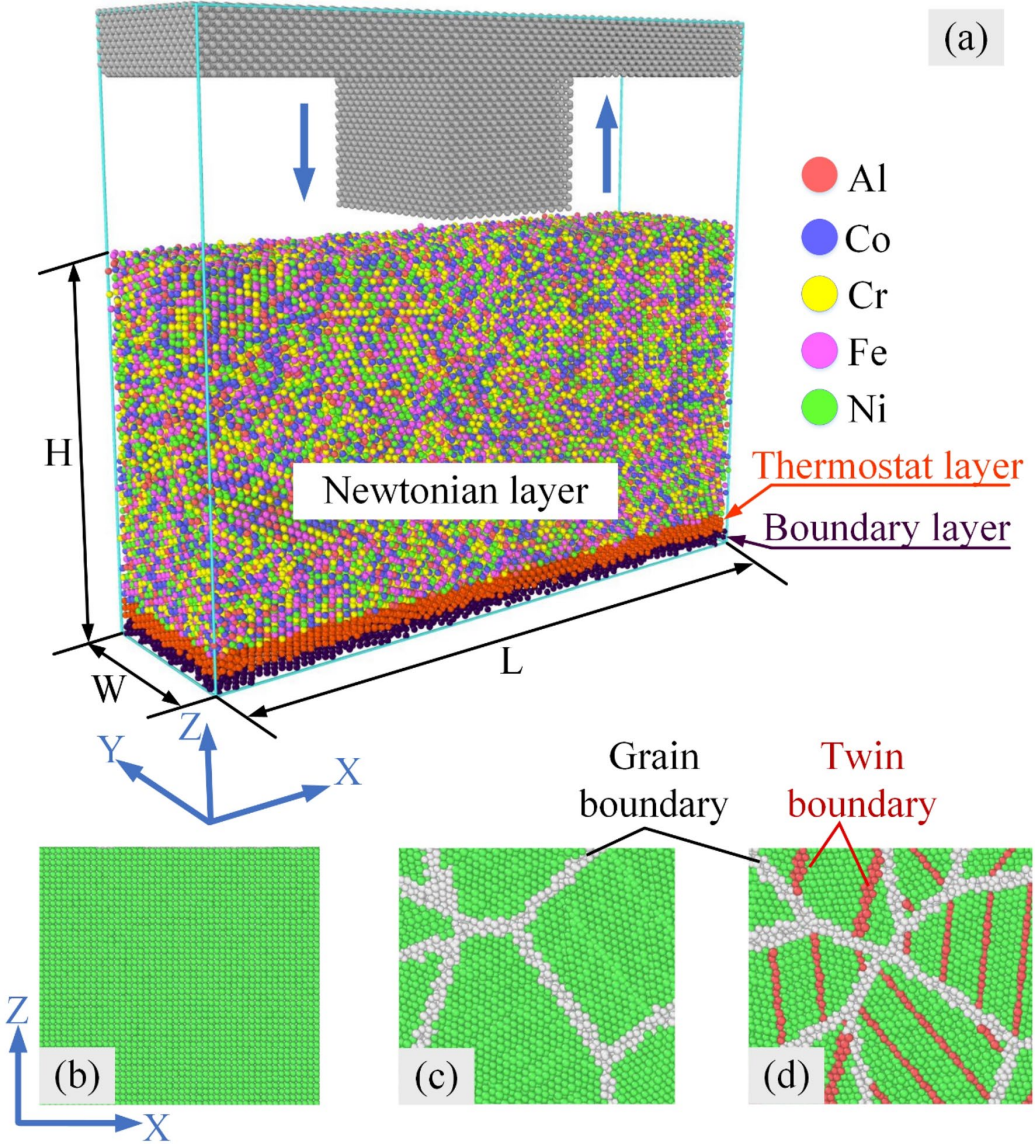
(**a**) The MD model of Al_x_CoCrFeNi HEA with different crystal structures during the nanoimprinting process; monocrystalline structure (**b**); polycrystalline structure (**c**); NT-polycrystalline structure (**d**).

The simulation model consists of AlCoCrFeNi HEA sample and punch, as shown in Fig. 1a. The samples are constructed through Atomsk software package^39^ (Version Beta-0.10.6 https://atomsk.univ-lille.fr/dl.php), and the polycrystalline structure is generated through the Voronoi tessellation technique^40^. The model size of 200 Å × 55 Å × 110 Å (L × W × H) containing between 113,088 and 114,601 atoms is generated to study the deformation behavior of FCC Al_x_CoCrFeNi HEAs with various crystal structures during the nanoimprinting process. The lattice constant of the FCC phase is 3.525 Å, which obtains from previous studies^41,42^. The punch in the diamond lattice structure consists of about 57,888 atoms, and it is loaded along the Z direction.

**Figure 2:**
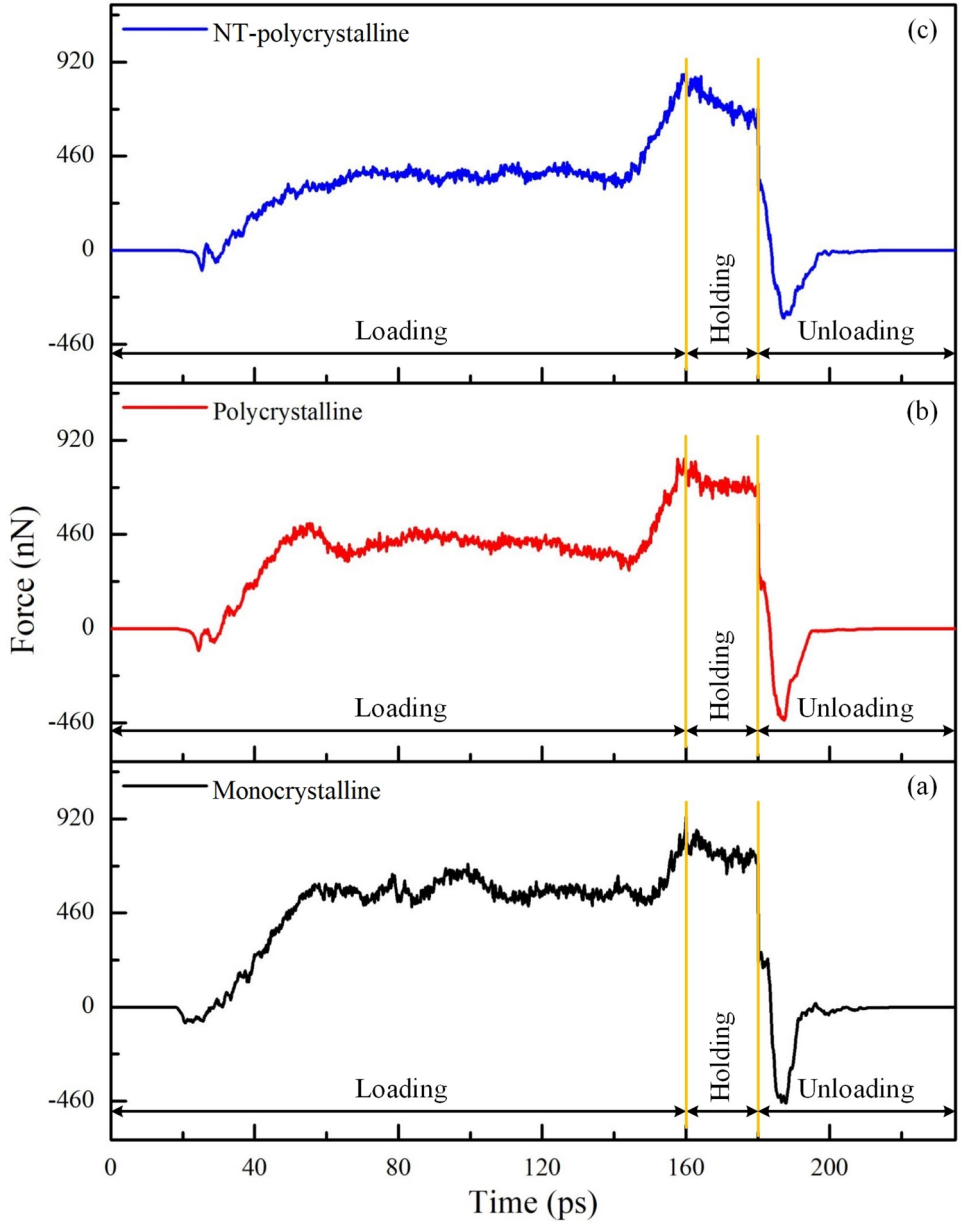
The imprinting force curves of Al0.5 HEA during the nanoimprinting for various crystal structures: monocrystalline (**a**); polycrystalline (**b**); NT-polycrystalline (**c**).

Three different crystal structures are applied, including monocrystalline, polycrystalline, and NT-polycrystalline, to explore the influence of crystal structure on the mechanistic characteristic and microstructure development of Al0.5 HEA under the nanoimprinting. The imprinting force curves of Al0.5 HEA during the nanoimprinting for different crystal structures are shown in Fig. 2. The force curves correspond to the three phases of the nanoimprinting: loading, holding, and unloading phases. During the loading phase, the first observation manifests that a van der Waals attractive force, expressed as negative force values in the curves, between the punch and sample has appeared before they come into complete contact. In the period from 30 to 60 ps, the loading forces increase rapidly as the penetration depth increases. Then the imprinting force is relatively stable to approximately 150 ps. Finally, the force curves tend to increase strongly again at the last loading stage. This is due to the overfilling of the material in the mold leading to a drastic increase in the pressure exerted on the mold, resulting in the loading force increases during this period. The evolution of loading force is similar to that of previous studies during nanoimprinting. For example, Wu et al.^43^ and Fang et al.^44^ revealed that the loading force curves are increased gradually as increasing the imprinting depth during the first loading phase, and the loading forces increase significantly with a further increase of the imprinting depth due to overfilling. Additionally, Fig. 2 reveals that the largest imprinting forces are 830.20 nN, 866.92 nN, and 930.38 nN for the polycrystalline, NT-polycrystalline, and monocrystalline specimens, respectively. The order of the highest imprinting force for various crystal structures is consistent with the previous investigations, such as the report by Doan et al.^45^ and Li et al.^46^. There is a difference in the greatest imprinting force due to the released stress induced by the slide and twist of grain boundaries (GBs) in the polycrystalline structure, resulting in a decrease in the loading force of the polycrystalline sample. While the increase in the imprinting force of the NT-polycrystalline specimen is due to the existence of TBs, leading to enhanced grain stability. Hence, the results show that GBs and TBs clearly inhibit the movement of punch in the microscopic crystal structures. The holding phase maintained between 160 and 180 ps indicates a decrease in the imprinting force because the adjustment of the atomic position during the holding phase causes a decrease in the strain energy stored in the substrate. At the beginning of the unloading phase, the imprinting forces drop suddenly to large negative values due to the adhesion phenomenon between the punch and the workpiece^47^. The results also show that the adhesion phenomenon is the strongest occurrence with monocrystalline structure. Ultimately, the imprinting force returns to zero when the punch completely withdraws from the substrate.

**Figure 3:**
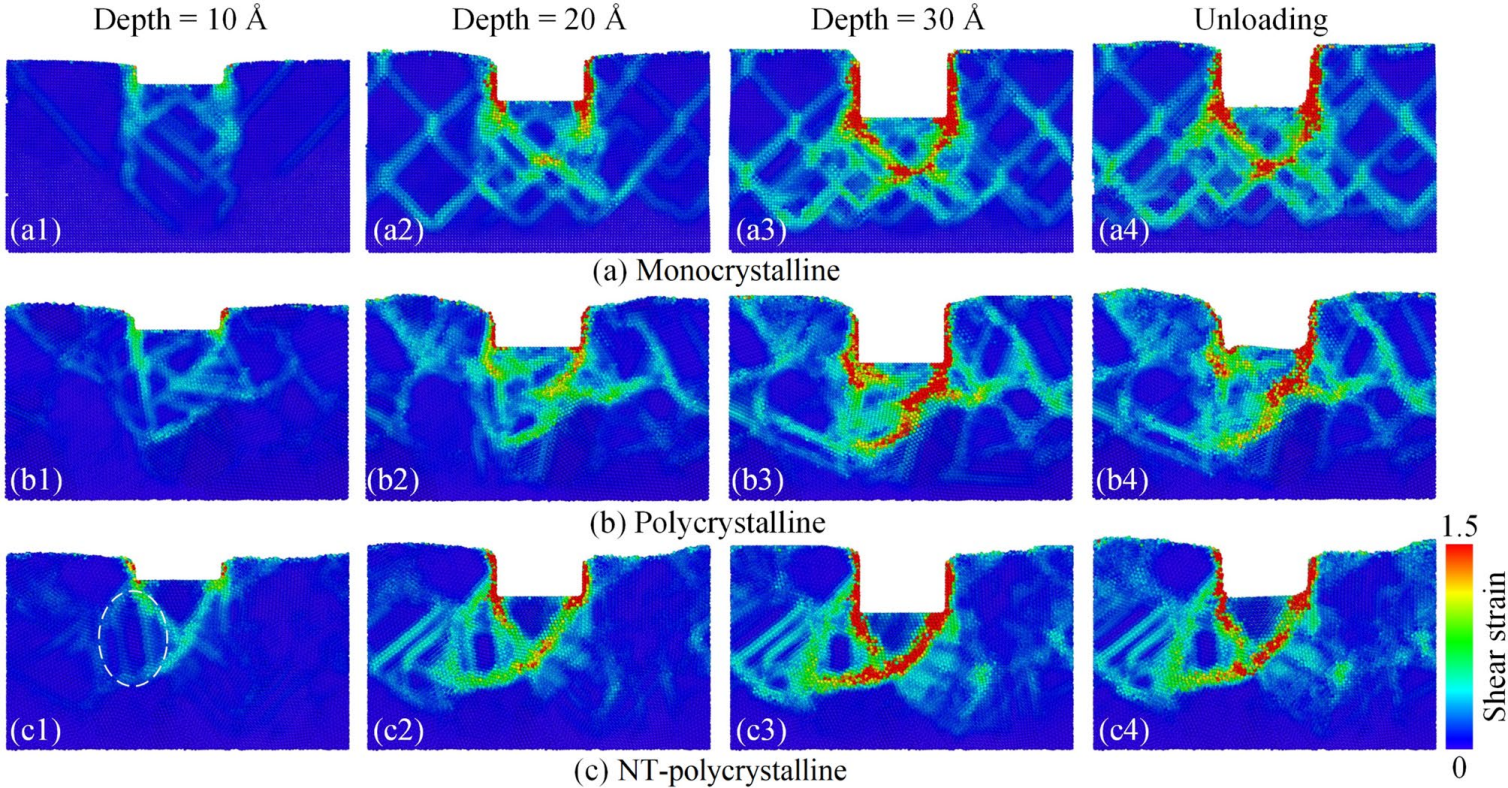
The cross-section of atomic shear strain distributions at the various imprinting depths and unloading stage of the Al0.5 HEA for different structures: monocrystalline (**a1**–**a4**); polycrystalline (**b1**–**b4**); and NT-polycrystalline (**c1**–**c4**).

To further investigate the effect of the crystal structure on the mechanistic characteristics of Al0.5 HEA during nanoimprinting, atomic-scale analysis consisting of the atomic shear strain and local stress is conducted. Commonly, dislocation slip is the control mechanism of the deformation process for metallic materials. So a large number of dislocations and phase transformation are observed during the nanoimprinting, which leads to irreversible plastic deformation behavior. Figure 3 indicates the cross-section of atomic shear strain distributions at the various imprinting depths and unloading stage of the Al0.5 HEA for different crystal structures. The atoms of the workpiece are painted according to the von Mises shear strain value. When the punch begins to imprint on the substrate at an imprinting depth of 10 Å, the atomic shear strain value of the substrate atoms underneath the punch increases, and the shear strain around the punch is greater than that in other areas of the sample. The atomic region in high shear strain values increases with increasing the penetration depth, accompanied by high shear strain intensity also increases significantly as shown in Fig. 3a2–c2. As the imprinting depth increases further to 30 Å, the number of atoms with high shear strain (red atoms) constantly increases from both sides of the punch extending into the substrate interior. At the unloading stage, the number of atoms subjected to large shear strain indicates a decreased trend compared with the atomic state at an imprinting depth of 30 Å because the atoms in the substrate are relaxed. For monocrystalline structure, the shear strain shows symmetry on both sides of the punch. In contrast, the polycrystalline and NT-polycrystalline structures display uneven plastic deformation because the grain and twin boundaries impede the evolution of shear strain. Different from monocrystalline structure, the shear strain mainly propagates into the material along GB of polycrystalline structures. The shear strain also spreads along TB of NT-polycrystalline as exhibited in the dashed ellipse. Apparently, the propagation of shear strain into the material interior for monocrystalline structure is deeper and wider than in the NT-polycrystalline and polycrystalline specimens. This is because the dislocation and stacking fault come into contact with GBs and TBs, as well as interact with each other, resulting in the grain and twin boundaries obstructing the plastic deformation. Thus, the GBs and TBs have the ability to prevent the spread of shear strain into the grain interior.

**Figure 4:**
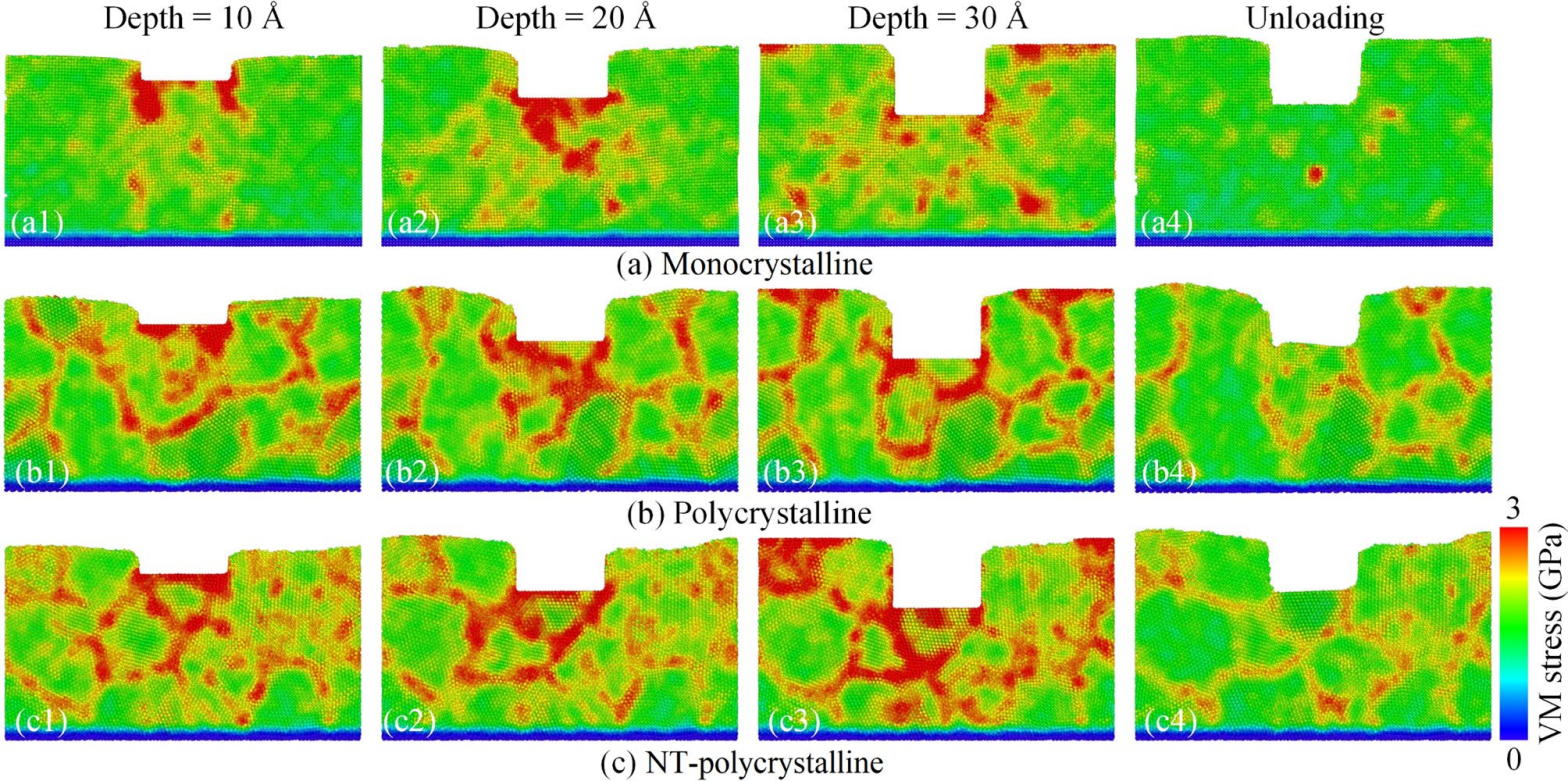
The cross-section of local stress dispersions of atoms at the various imprinting depths and unloading stage of the Al0.5 HEA for various crystal structures: monocrystalline (**a**); polycrystalline (**b**); and NT-polycrystalline (**c**).

Figure 4 indicates the cross-section of local stress dispersions of atoms at the various imprinting depths and unloading stage of the Al0.5 HEA for different crystal structures. The atoms of the workpiece are painted according to the von Mises (VM) stress value. At an imprinting depth of 10 Å, the stress concentration regions are primarily underneath the punch for monocrystalline structure. Meanwhile, the high local stress areas are not only below the imprinting tool but also on the GBs for polycrystalline and NT-polycrystalline structures. Moreover, the local stress near GBs is significantly greater than the stress of grain interior. The number of atoms with large local stress values increases with the increment in the imprinting depth to 20 Å and 30 Å. At the unloading stage, the atomic zones in large local stress are significantly decreased compared to the atomic state during the nanoimprinting process. In general, the area of high-stress concentration region of the NT-polycrystalline is greater than that of the polycrystalline, and both of these structures have a much greater local stress than the monocrystalline structure. Therefore, the existence of TBs in polycrystalline is improved the stability of grain, which leads to higher local stress in NT-polycrystalline sample than in polycrystalline structure.

**Figure 5:**
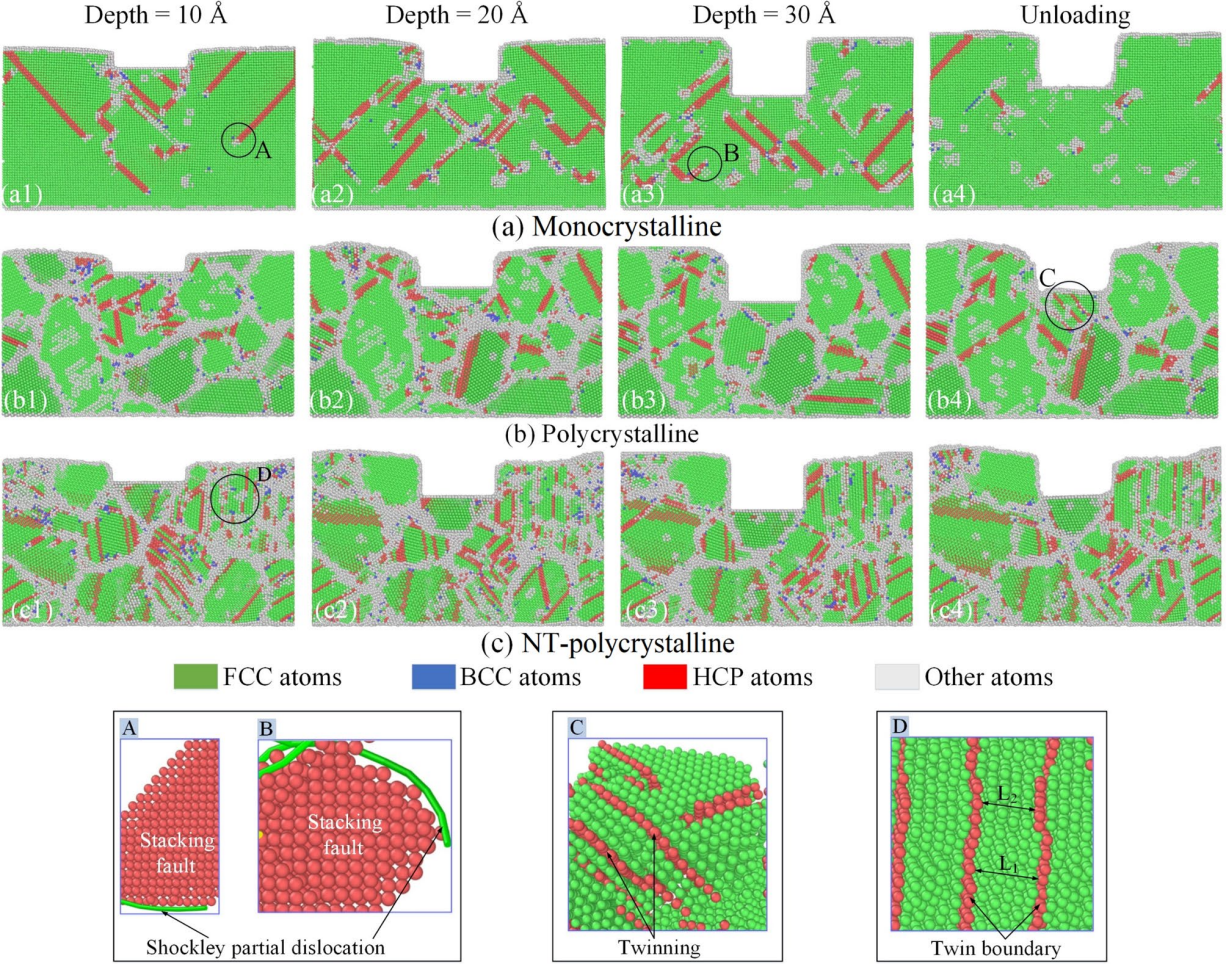
The evolution of microstructure at the various imprinting depths and the unloading stage of the Al0.5 HEA for different structures: monocrystalline (**a1**–**a4**); polycrystalline (**b1**–**b4**); and NT-polycrystalline (**c1**–**c4**).

Figure 5 displays the evolution of microstructure at the various imprinting depths and the unloading stage of the Al0.5 HEA for different structures. The atomic state is visualized through the CNA method. The partial dislocation, stacking fault, and twinning (region C) in the HCP structure are entirely recognizable in all specimens due to the complete release of stored elastic energy. It can be observed intuitively that the HCP phase increases as the imprinting depth increases from 10 to 20 Å, then it tends to decrease when the machining depth increases further to 30 Å in all models. The amorphous phase transformed from the original crystal structure is increased by increasing the penetration depth in all specimens. Besides, the BCC phase accounts for a small proportion compared to the other phases. At the unloading stage, the HCP, BCC, and amorphous structures tend to decrease vigorously, which means a strong recovery of the FCC phase. The microstructure evolution of samples during the nanoimprinting shows that the partial Shockley dislocations initiate to form underneath the punch when the imprinting tool approaches the surface of model. Then the movement of the leading partial dislocations will generate the stacking faults behind it, which is evident in regions A and B. It is found that the development of stacking faults and leading partial dislocations accompanies by amorphous structural formation. For the monocrystalline structure, the nucleation and movement of leading partial dislocations control the plastic deformation mechanism in the nanoimprinting, which can be observed through the dislocations moving almost across the entire workpiece. With polycrystalline and NT-polycrystalline samples, the GBs interfere with the motion of dislocation and stacking fault, accompanied by the twist and slide of GBs are occurred during the nanoimprinting. Moreover, the TB migration has occurred in the NT-polycrystalline workpiece, as displayed in Fig. 5c. The enlarged image of zone D displays the distance of TB changing from the original value *L*_1_ to the secondary value *L*_2_ at the penetration depth of 10 Å, which shows that the TB is spontaneous migration during the nanoimprinting.

**Figure 6:**
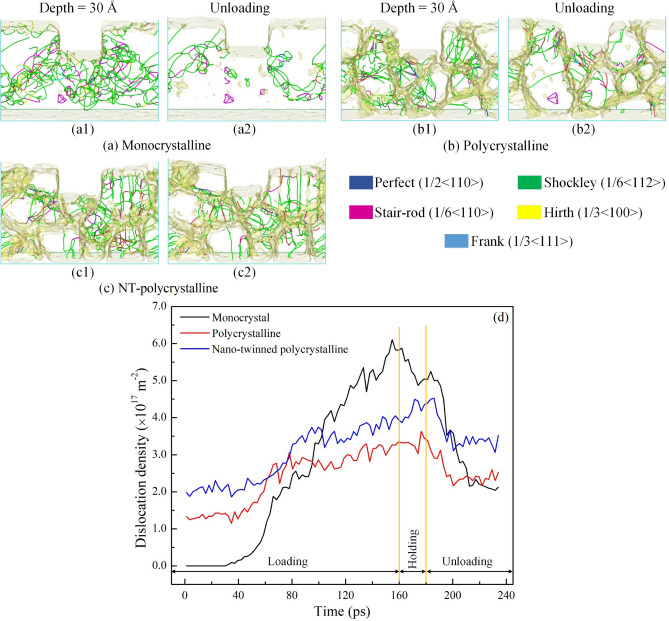
The distribution of identified dislocations at an imprinting depth of 30 Å and the unloading stage of the Al0.5 HEA for various crystal specimens: monocrystalline (**a**); polycrystalline (**b1**); and NT-polycrystalline (**c**). Evolution of the total dislocations density of the Al0.5 HEA during the nanoimprinting for different structures (**d**).

The evolution of the identified dislocations at an imprinting depth of 30 Å and the unloading stage of the Al0.5 HEA for various crystal specimens of monocrystalline, polycrystalline, and NT-polycrystalline is shown in Fig. 6a–c. For a monocrystalline structure, the dislocation emanates from the surface below the punch, and then the dislocation spreads to the entire specimen with an increase in imprinting depth, as shown in Fig. 6a1. For polycrystalline and NT-polycrystalline structures, the dislocations have a dense concentration on the GBs compared to the interior of the grain, and the dislocation distribution between the GBs is not homogeneous because of the distinctness in structure and orientation of GBs. Besides, the movement of dislocations meets and interacts with GB and TB leading to the dislocations being demolished or reacting with each other. The result leads to new dislocations formation in polycrystalline and NT-polycrystalline samples. As a result, the partial dislocations during the nanoimprinting of the monocrystalline are relatively complete and long in the entire sample. In contrast, the partial dislocations of the polycrystalline and NT-polycrystalline are relatively short. It is observed that the Shockley partial dislocation accounts for the majority of all types of dislocations. The Shockley, Stair-rod, and Hirth dislocations in the monocrystalline are significantly greater than those of polycrystalline and NT-polycrystalline. In comparison, the Perfect dislocations and other dislocations (curves in red) in the monocrystalline are smaller than the other two structures, which can be identified in Fig. 6a1–c1. At the unloading stage, the dislocations are greatly reduced especially in the monocrystalline sample.

The evolution of the total dislocations density of the Al0.5 HEA during the nanoimprinting for different crystal structures is given in Fig. 6d. The dislocation densities of the polycrystalline and NT-polycrystalline do not start from zero as monocrystalline at the beginning of the nanoimprinting. This phenomenon is due to the initial existence of the dislocations on the GB due to the influence of the annealing process of specimens^48,49^. During the loading stage, the dislocation density curves strongly increase for three different crystal structures. The dislocation density curves tend to increase during the holding stage for the polycrystalline and NT-polycrystalline, while the dislocation density curve is decreased for the monocrystalline. The dislocation density of the monocrystalline is greater than the other two structures in both loading and holding phases. This implies that the evolution of dislocations is hindered by the GB. The strength of metallic materials depends on the difficulty of dislocation movement^50,51^, and the deformation mechanism of polycrystalline depends on the activity of GB^52^. The appearance of GBs in the polycrystalline and NT-polycrystalline substrates reduces the number of dislocation, which also leads to a decrease in the strength of the material compared to the monocrystalline structure. Meanwhile, the presence of TBs in NT-polycrystalline improves material strength compared to the polycrystalline structure. Besides, the HCP phase is more formed in the NT-polycrystalline, resulting in a greater dislocation density of NT-polycrystalline than in the polycrystalline structure. The dislocation density curves reduce for all models during the unloading stage, especially the monocrystalline structure has drastically decreased. Therefore, it indicates that the GB and TB play a noticeable role in the evolution of dislocation for the polycrystalline and NT-polycrystalline samples.

**Figure 7:**
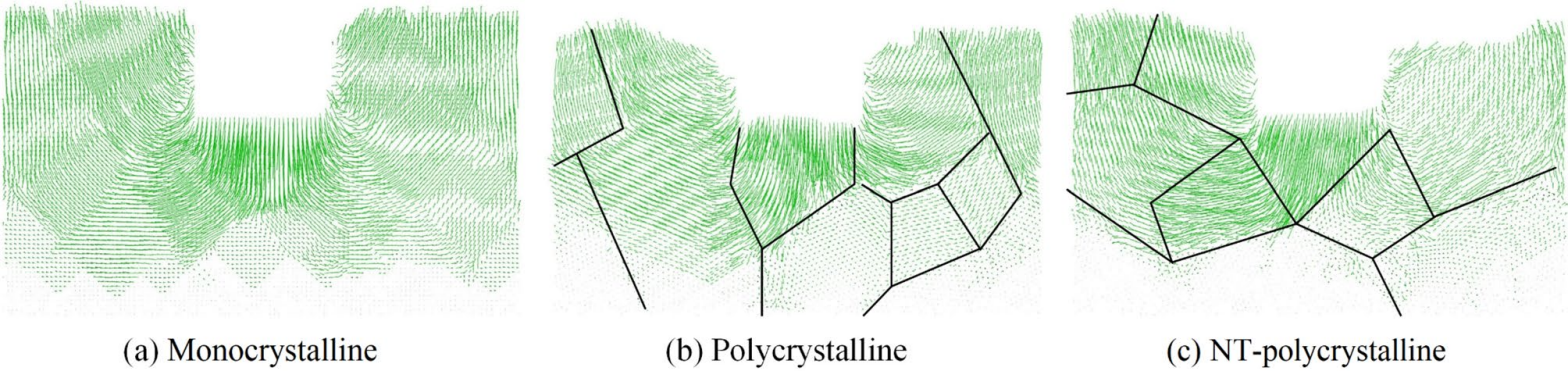
The cross-sectional view of the displacement vectors of atoms under the nanoimprinting at the unloading stage of the Al0.5 HEA for various crystal structures: monocrystalline (**a**); polycrystalline (**b**); and NT-polycrystalline (**c**).

Figure 7 shows the cross-sectional view of the displacement vectors of atoms under the nanoimprinting at the end of the unloading stage of the Al0.5 HEA for various crystal structures. The displacement vector can be determined as the change in the position of atoms during nanoimprinting. It can be observed that the motion of atoms has occurred at the underneath of the punch, then spreads to both sides to form the atomic displacement regions in all structures. The direction of the atomic displacement depends on the pressure during nanoimprinting. The area below the punch is under compressive stress, so the atoms move downwards. While the regions on both sides of the punch are subjected to tensile stress, leading to the atoms move upward. For the monocrystalline structure, the movement of atoms describes the degree of symmetry on both sides of the punch. For the polycrystalline and NT-polycrystalline samples, the displacement vectors of atoms exhibit asymmetry because of the presence of GBs and the random orientation of grains. It shows the GB blocking and changing the displacement directions of the atoms, which suggests that the GB is the barrier to atomic motion. The result leads to the asymmetric distribution of the motion vectors of atoms. In conclusion, it indicates that the GBs and TBs play a controlling role in the deformation and reinforcement mechanism of AlCoCrFeNi HEA.

### Influence of alloy composition

**Figure 8:**
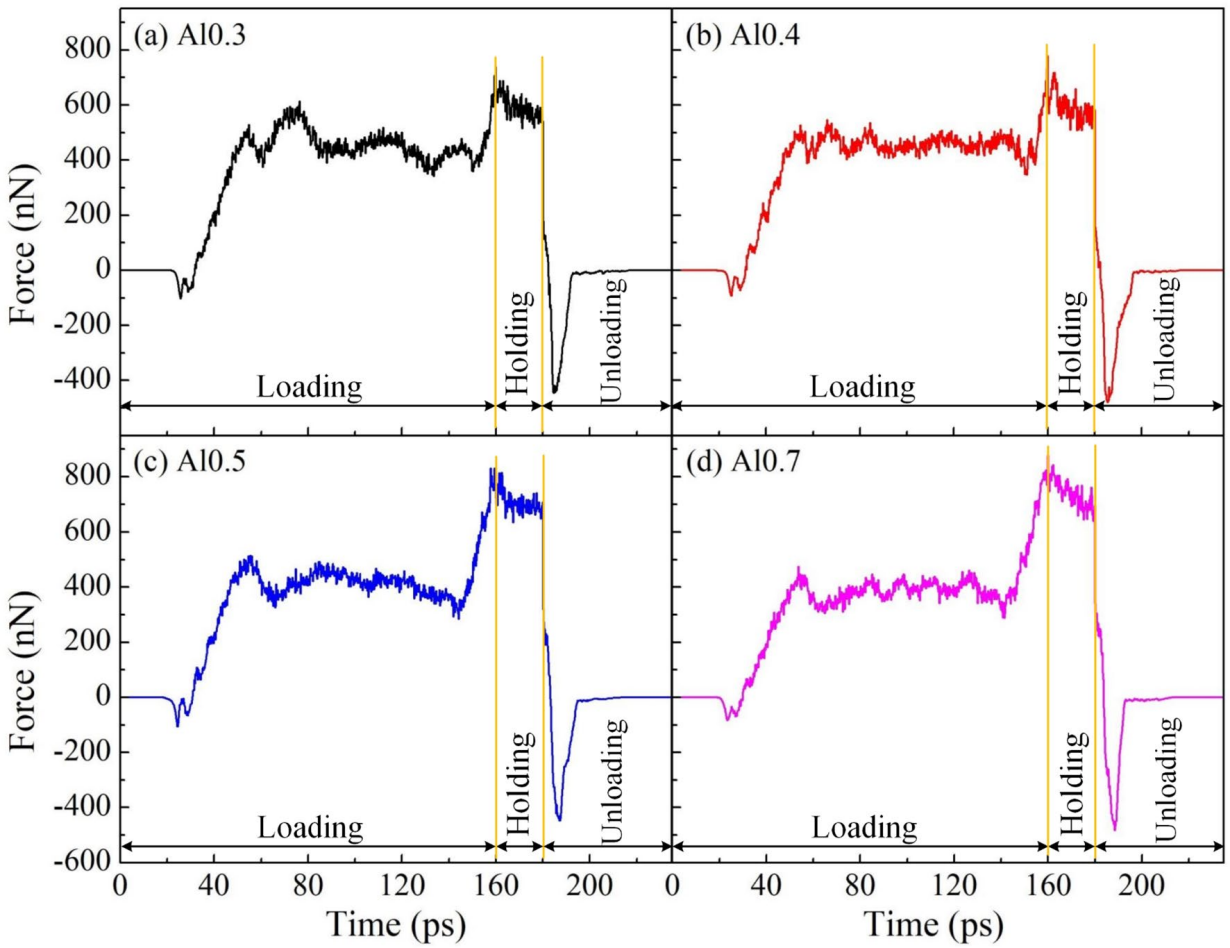
The force–time diagrams for polycrystalline specimens during the nanoimprinting process with various alloy compositions: Al0.3 (**a**); Al0.4 (**b**); Al0.5 (**c**); and Al0.7 (**d**).

Four different alloy compositions of Al_7.2_Co_23.2_Cr_23.2_Fe_23.2_Ni_23.2_ (Al0.3), Al_9.2_Co_22.7_Cr_22.7_Fe_22.7_Ni_22.7_ (Al0.4), Al_11.0_Co_22.2_Cr_22.2_Fe_22.3_Ni_22.3_ (Al0.5), and Al_11.6_Co_22.1_Cr_22.1_Fe_22.1_Ni_22.1_ (Al0.7) are employed to examine the effects of alloy composition on the mechanistic reaction of polycrystalline structure during the nanoimprinting. Figure 8 shows the force–time diagrams for polycrystalline specimens during the nanoimprinting process with various alloy compositions. The imprinting force development shows the similarities as discussed above in the loading phase. The imprinting force curves increase rapidly in the initial stage before entering the stable force phase for a long time. The loading forces increase sharply again with a further increase of penetration depth, which is associated with increasing the contact area and overfilling between the substrate and the punch, resulting in increased imprinting force. The maximum imprinting forces are 736.20, 778.04, 830.20, and 877.67 nN corresponding to alloy compositions of Al0.3, Al0.4, Al0.5, and Al0.7, respectively. It reveals that there is a positive correlation between the Al content and maximum load that the largest loading forces increase with increasing the Al content in Al_x_CoCrFeNi HEA. The result is a good consensus with previous studies as reports by Wang et al.^24^ and Lv et al.^53^. They indicated that the hardness of Al_x_CoCrFeNi HEA in the FCC structure increases with the increase in the Al proportion. The imprinting forces decrease gradually at the holding phase. The forces then quickly reduce to negative values in all diagrams due to the appearance of the adhesion phenomenon between the substrate and the punch when the punch has retracted from the workpiece during the unloading phase.

**Figure 9:**
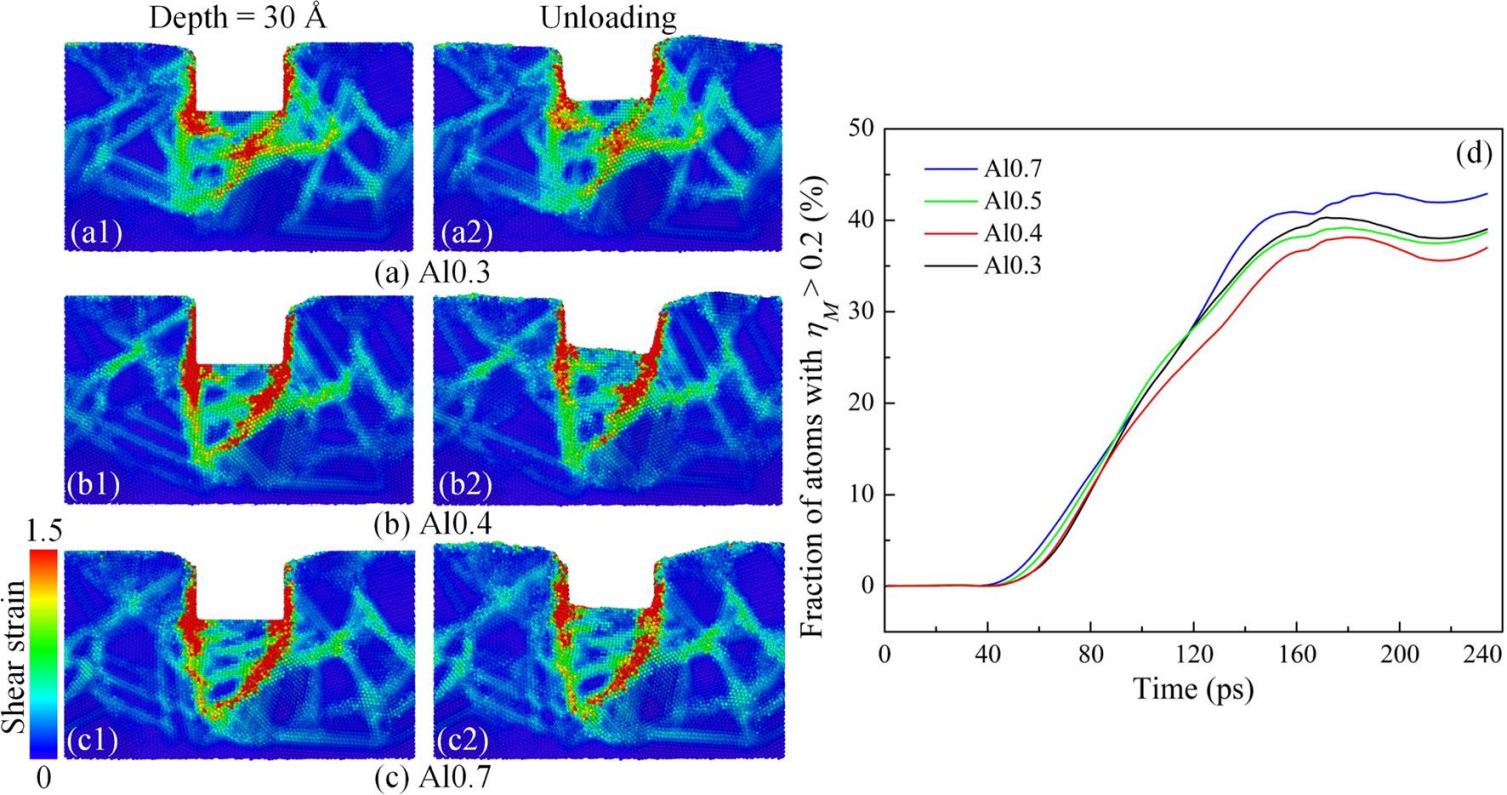
The cross-section of atomic shear strain distributions for the polycrystalline structure of the Al_x_CoCrFeNi HEA at an imprinting depth of 30 Å and the unloading stage with different alloy compositions: Al0.3 (**a**); Al0.4 (**b**); and Al0.7 (**c**). The fraction of atoms with atomic shear strain value *η*_*M*_ greater than 0.2 (**d**).

Figure 9a–c displays the cross-section of atomic shear strain distributions for the polycrystalline structure of the Al_x_CoCrFeNi HEA at an imprinting depth of 30 Å and the unloading stage with different alloy compositions. The atoms of substrate have a color matching the value of von Mises shear strain. All the snapshots for Al0.5 HEA are shown in Fig. 3b3,b4. It is found that the deformation mechanism of specimens with various alloy compositions is similar. Specifically, the atomic regions around the punch are subjected to higher shear strain values for all samples, where the pressure is the largest during the nanoimprinting. Additionally, the strain propagates into the workpiece through GBs, represented by the GBs with large shear strain values. This suggests that the GB has a great influence on the deformation of HEA with the change in alloy content. Although the deformation mechanism is analogous, the shear strain level is different between specimens with various alloy compositions, which is expressed by the number of atoms subjected to high shear strain during the nanoimprinting. To further analyze the atomic responses during the nanoimprinting, the fraction of atoms with atomic shear strain value *η*_*M*_ greater than 0.2 is determined and presented in Fig. 9d. The threshold value of 0.2 is chosen based on the distribution of the von Mises shear strain for all the atoms in the sample. All curves remain zero at the preliminary phase, which corresponds to the elastic phase during deformation. Then all curves are increased linearly and go sideways after 160 ps. The result exhibits that the Al0.7 HEA specimen with the highest Al content undergoes the greatest plastic shear strain, while the Al0.4 HEA sample has the lowest number of atoms subjected to great shear strain. Therefore, the number of atoms subjected to high shear strain is insensitive to changes in the alloy composition.

In addition, the microstructure snapshots at an imprinting depth of 30 Å and the unloading stage for the polycrystalline structure of the Al_x_CoCrFeNi HEA with different alloy compositions are displayed in Supplementary Fig. 1a-c. The results indicate that the evolution of microstructure is analogous with different alloy compositions in which the slide and twist of GB is the control mechanism in the deformation. It can be found that the dislocation, stacking fault, and twinning appear in all workpieces during the loading and unloading stages, and they are suppressed by the GBs. At the unloading stage, it points to a strong recovery of the FCC structure, while all other structures significantly decrease in proportion. The development of the total dislocation density of the Al_x_CoCrFeNi HEA during the nanoimprinting with different alloy compositions displays in Supplementary Fig. 1d. The dislocation densities tend to increase during both phases of loading and holding, whereas the dislocation density decreases during the unloading phase in all cases. The result also shows that the dislocation density indicates a decreasing trend with an increase in Al content during the loading and holding stages. The inverse correlation between maximum loading force and dislocation density is revealed with the change of Al content in HEA. The activities of partial dislocations and GBs dominate the plastic deformation and lead to the strain hardening in the polycrystalline^54^. Many dislocation movements on the active slip systems can locally result in small strain hardening rate. In other words, the lower dislocation density induces a greater strain hardening rate, leading to a higher loading force. Therefore, the evolution of deformation and structure is greatly influenced by alloy components in Al_x_CoCrFeNi HEA.

### Influence of grain size

**Figure 10:**
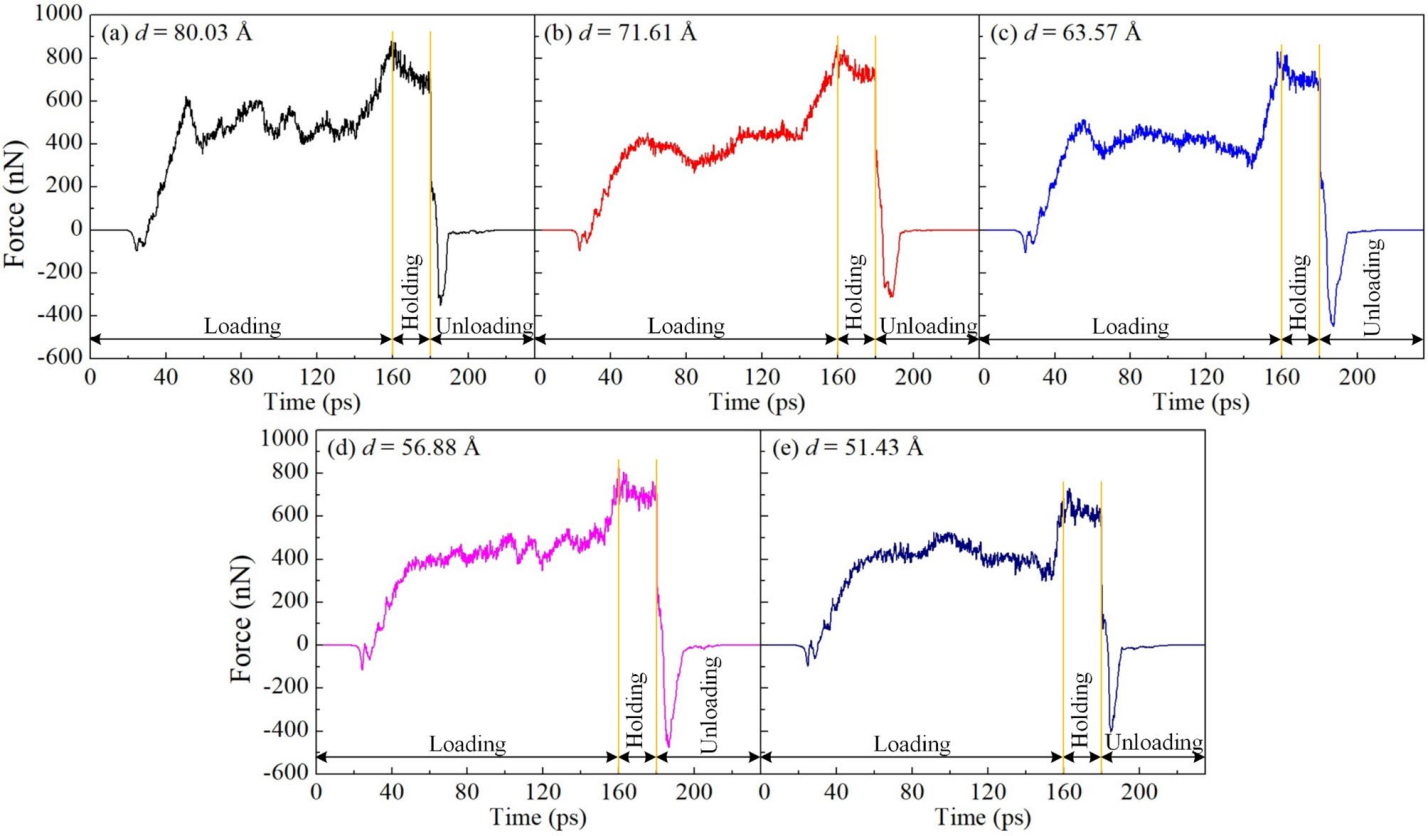
The force–time diagrams for polycrystalline Al0.5 HEA specimens during the nanoimprinting process with various grain sizes: 80.03 Å (**a**); 71.61 Å (**b**); 63.57 Å (**c**); 56.88 Å (**d**); and 51.43 Å (**e**).

In this section, five polycrystalline samples with various grain sizes of 80.03, 71.61, 63.57, 56.88, and 51.43 Å are prepared to probe the effect of grain sizes on the mechanistic characteristics of the Al0.5 HEA during nanoimprinting. Figure 10 shows the force–time diagrams for polycrystalline Al0.5 HEA specimens during the nanoimprinting process with various grain sizes. Generally, the loading force increases with the increase of the imprinting depth. The results reveal that the highest loading forces are 879.45, 858.55, 830.20, 826.86, and 730.07 nN corresponding to grain sizes of 80.03, 71.61, 63.57, 56.88, and 51.43 Å, respectively. It shows a positive relationship between the grain size and the largest loading force. This phenomenon reveals that the GB clearly relates to the softening of the polycrystalline structure. The smaller grain size samples have more GBs, leading to the loading force is reduced with the decrease in the grain size. The reduction of grain size improves the strength of the material indicating a Hall–Petch relation^47^. However, many previous studies demonstrate that grain refinement below a critical grain size induces a decrease in the material strength, which manifests the reverse Hall–Petch relation. The reason is that the main deformation mechanism has shifted from the dislocation actions to the activities of the grain boundary^55^. Chen et al.^56^ investigated the CoNiFeAlCu HEA under the tensile test. They explored that the reserve Hall–Petch relation is found when the grain size is below 12.1 nm, which the yield stress has significantly decreased with the decrease of particle size. Zhang et al.^57^ investigated the mechanical behavior of FeNiCrCoCu HEA under the tensile test. They reported that the flow stress is reduced from 3.60 GPa to 3.21 GPa with the reduction of particle size from 12.5 and 5 nm, and the result revealed that the reverse Hall–Petch relation is observed. Accordingly, the reverse Hall–Petch relation is recognized in the current study.

**Figure 11:**
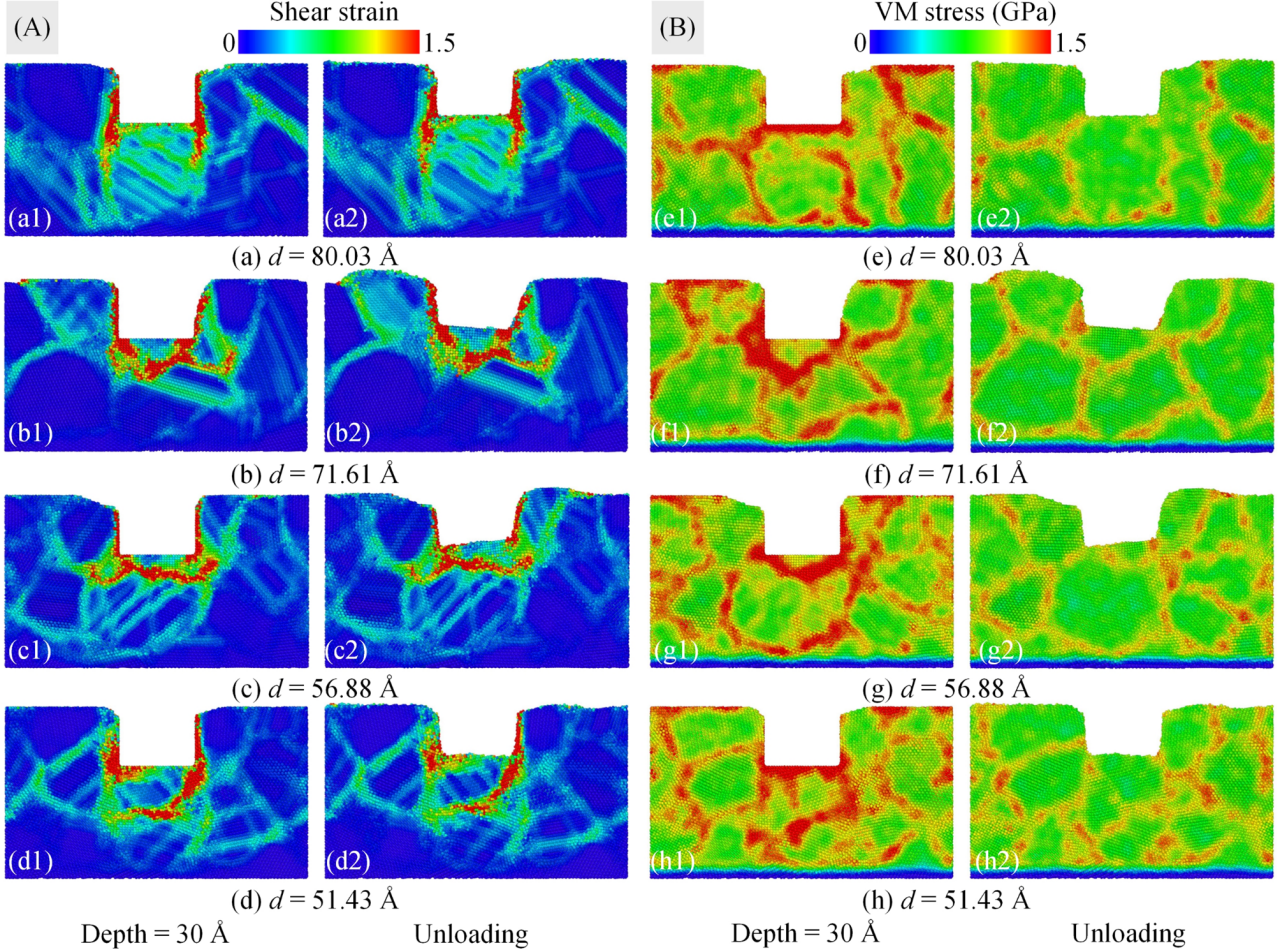
The cross-section of atomic shear strain distributions (**a**–**d**) and the local stress dispersions of atoms (**e**–**h**) for the polycrystalline structure of the Al0.5 HEA at an imprinting depth of 30 Å and the unloading stage with various grain sizes: 80.03 Å (a); 71.61 Å (**b**); 56.88 Å (**c**); and 51.43 Å (**d**).

Figure 11 discloses the cross-section of atomic shear strain distributions (a-d) and the local stress dispersions of atoms (e–h) for the polycrystalline structure of the Al0.5 HEA at an imprinting depth of 30 Å and the unloading stage with various grain sizes. The snapshots of stress and strain for a grain size of 63.57 Å exhibit in Figs. 3b3,b4, 4b3,b4. It is observed that the deformation becomes more severe with the reduction of grain size, indicated by the shear strain in material is increased with a decrease in grain size during the nanoimprinting at a penetration depth of 30 Å as shown in Fig. 11a1–d1. The result of deformation is the distribution of gradient strain from the machined position to the inside of the substrate, and the sample with a smaller grain size has more GBs leading to stronger gradient strain. Therefore, the specimen with a smaller grain size has greater shear strain^58^. The von Mises stress distributions of polycrystalline samples show that the larger stress concentration zone is generated with the smaller the grain size, as displayed in Fig. 11e1–h1. The results also reveal that the stress concentration zones focus primarily on GBs and the surrounding areas of GBs. Hence, the dense GB specimen bears strong deformation propagation into the substrate along the GBs, accompanied by a GBs expansion. At the unloading state, the same deformation behavior occurs that the stress and strain are greatly reduced compared to the loading stage, which indicates in Fig. 11a2–d2,e2–h2.

**Figure 12:**
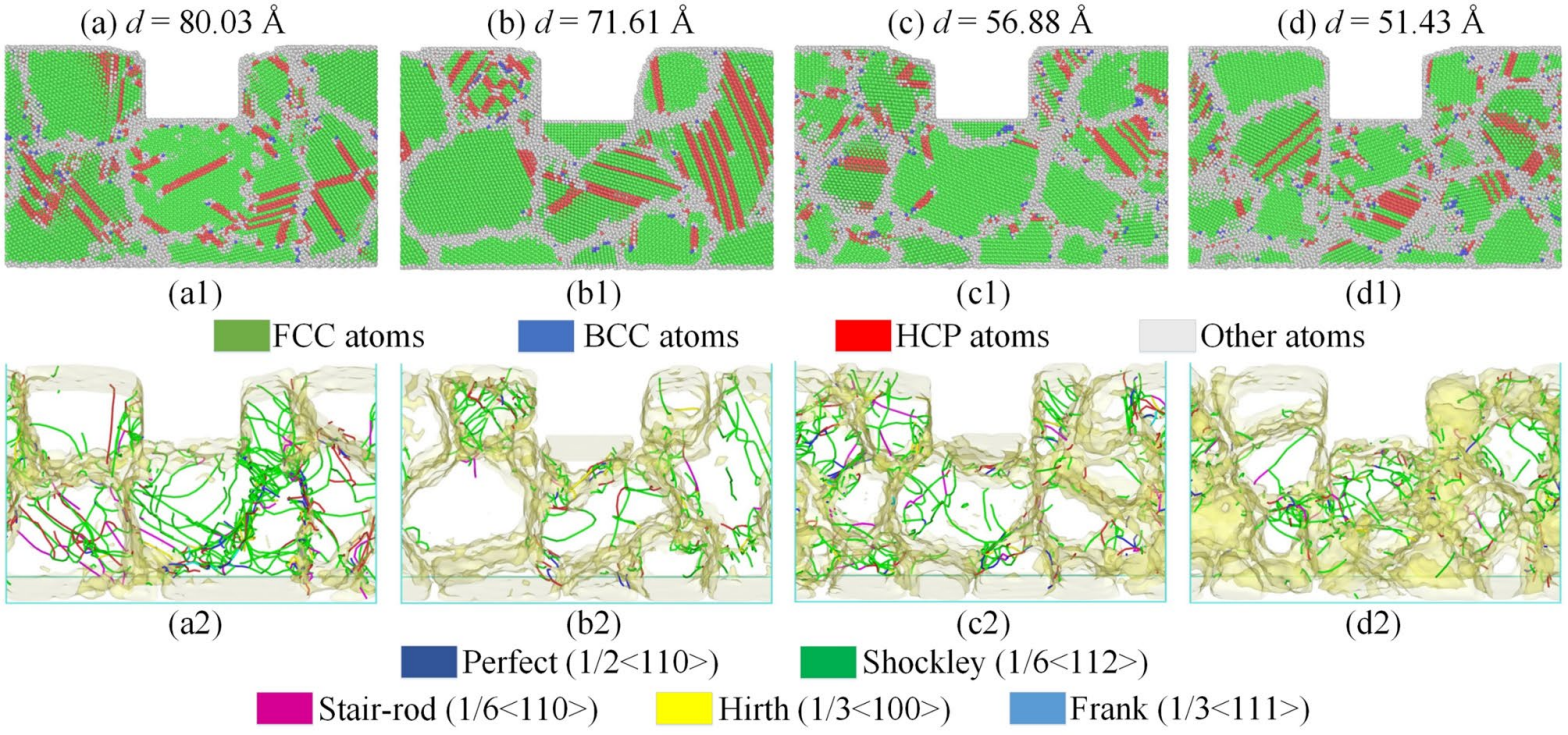
The microstructure snapshots (**a1**–**d1**) and distribution of the identified dislocations (**a2**–**d2**) at an imprinting depth of 30 Å for polycrystalline samples of the Al0.5 HEA with various grain sizes: 80.03 Å (**a**); 71.61 Å (**b**); 56.88 Å (**c**); and 51.43 Å (**d**).

Figure 12 exposes the microstructure snapshots (a1–d1) and distribution of the identified dislocations (a2–d2) at an imprinting depth of 30 Å for polycrystalline samples of Al0.5 HEA with various grain sizes. The deformation behavior clearly depends on the grain size with the stacking faults and leading partial dislocations moving across the grains, which can be noticed in Fig. 12a1–d1. GB is considered a factor to prevent the movement of dislocation and stacking fault. For specimens with more GB lead to more interference. As a result, the deformation becomes intense with decreasing the grain size. During the nanoimprinting, the dislocations may interact with each other to create the dislocation network, or they can interact with GB and be absorbed resulting in GB expansion, which can be observed in Fig. 12a2–d2. The result shows that the dislocation curves have a reduced length with decreasing grain size due to the suppression of dense GB. Furthermore, numerous Shockley partial dislocations are present in all workpieces, while Hirth and Frank dislocations are the least in all types of dislocations.

### Influence of twin boundary distance

**Figure 13:**
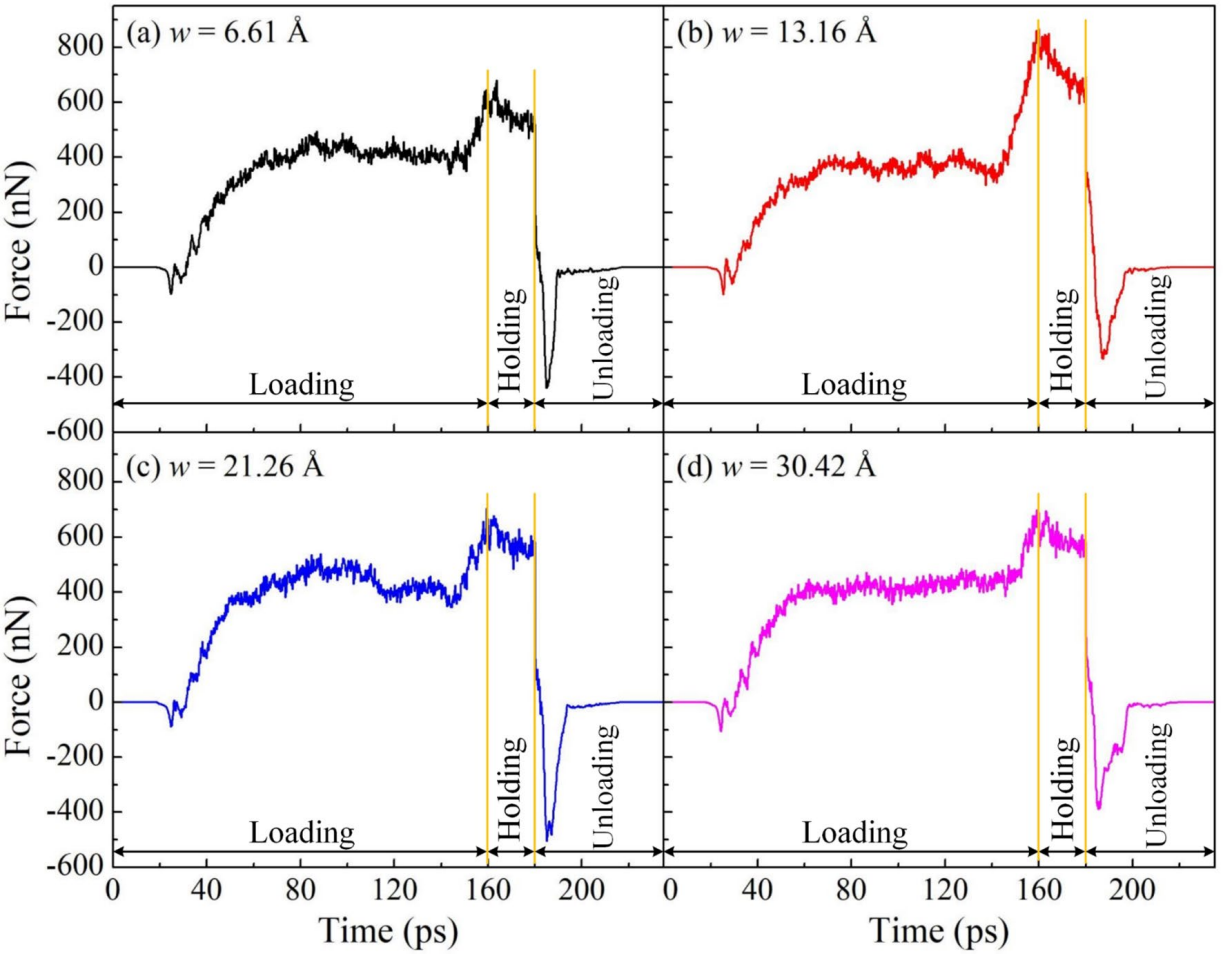
The force–time diagrams for NT-polycrystalline workpieces during nanoimprinting with various TB distances: 6.61 Å (**a**); 13.16 Å (**b**); 21.26 Å (**c**); and 30.42 Å (**d**).

Several studies have found that TB plays a significant role in the deformation behavior of HEAs, such as Tian et al.^59^ and Qi et al.^60^. The remarkable influence of TB on the deformation behavior of HEA is shown based on the results of impact load, deformation behavior, and microstructure evolution. The plastic deformation mechanism of NT-polycrystalline is governed by the suppression of dislocation by TB, the TB migration, and the formation of dislocation and stacking fault at TB. To probe the influence of various TB distances on the mechanical characteristics of NT-polycrystalline Al0.5 HEA under the nanoimprinting, four various TB distances of 6.61, 13.16, 21.26, and 30.42 Å are chosen. Figure 13 exhibits the force–time diagrams for NT-polycrystalline workpieces during nanoimprinting with various TB distances. The imprinting force is zero due to the gap between the sample and the punch during the first segment of the loading phase and the end of the unloading phase. The loading force is increased after the punch contacts completely the substrate. The result provides that the maximum loading forces for the TB distances of 6.61, 13.16, 21.26, and 30.42 Å are 679.90, 866.92, 704.69, and 698.85 nN, respectively. It shows that the peak loading forces increase with the decrease of TB distance from 30.42 to 13.16 Å, which discloses the Hall–Petch relation. It clearly indicates that the existence of more TBs in the polycrystalline structure enhances the strength of the material. Meanwhile, too many TBs in the polycrystalline structure are clearly related to the material softening, leading to the maximum loading force being reduced with the further decrease of TB distance from 13.16 to 6.61 Å. This satisfies the reverse Hall–Petch relation. The result is consistent with recent studies^61,62^. For example, Tian et al.^63^ investigated the influence of TB spacing on the mechanical behavior of polycrystalline TiAl under the tensile and compression tests. They showed that the mean flow stress reaches the highest value at a critical TB spacing of 21.6 Å, which shows the Hall–Petch relation. When TB spacing is less than 21.6 Å, the mean flow stress reduces with a further reduction of TB spacing, and the reverse Hall–Petch relation occurs. Accordingly, TB distance is significantly affected the mechanical property of NT-polycrystalline.

Furthermore, Supplementary Fig. 2 provides the cross-section of atomic shear strain distributions (a1–c1) and the local stress distributions of atoms (a2–c2) for the NT-polycrystalline structure of the Al0.5 HEA at an imprinting depth of 30 Å with various TB distances. All the snapshots for TB distance of 13.16 Å are shown in Figs. 3c3 and 4c3. The larger strain and stress concentration regions are primarily focused around the punch, as well as on GBs and the adjacent zones. The strain and stress fields interact not only with GBs but also with TBs for the NT-polycrystalline structure. This is demonstrated through the difference in shear strain and local stress distributions of the samples with various TB distances during nanoimprinting at a penetration depth of 30 Å. As a result, the number of atoms subjected to high shear strain is the greatest for the specimen with a TB distance of 6.61 Å, indicating that the specimen with the smallest TB distance is more severely deformed than the other cases. This implies that TB also plays a remarkable role in inhibiting the expansion of shear strain and local stress to the interior of the substrate.

**Figure 14:**
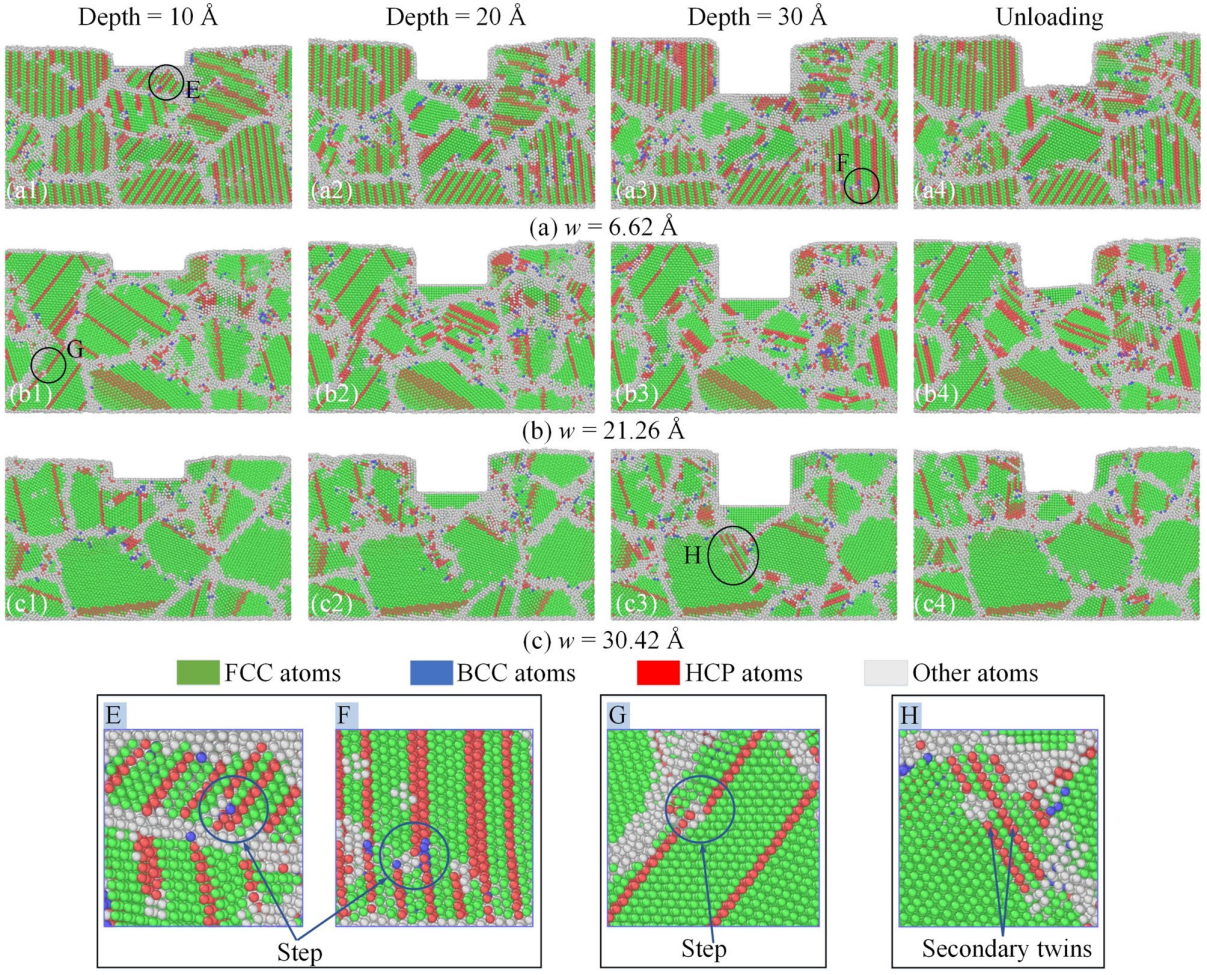
The microstructure snapshots at the different imprinting depths and the unloading stage for the NT-polycrystalline structure of the Al0.5 HEA with various TB distances: 6.61 Å (**a**); 21.26 Å (**b**); and 30.42 Å (**c**).

The microstructure snapshots at the different imprinting depths and the unloading stage for the NT-polycrystalline structure of the Al0.5 HEA with various TB distances are displayed in Fig. 14. Different from monocrystalline structure, the combined response of TBs and GBs controls the plastic deformation mechanism of the NT-polycrystalline. It is observed that the TBs impede the motions of the dislocations and stacking faults, and the migrations of TBs are noticed in the specimens. The dislocation nucleation at the GB-TB junctions can also greatly influence the plastic deformation, especially with a small TB distance. For a small TB distance of 6.62 Å, the migration of TB in the grains adjacent to the punch is observed at an imprinting depth of 10 Å with the obvious steps in TBs beneath the punch, as shown in zone E. As the loading continues to depths 20 Å and 30 Å, the TBs far from the punch migrate to create steps in the TBs as indicated in region F, and the TB migration is still led by the leading partial dislocation. The migration of TBs does not show clearly for a TB distance of 21.26 Å, while the inhibition of TBs and the formation of stacking faults in the grains around the punch are found. However, the migration of TBs is observed in the grains far from the punch at a depth of 10 Å, which shows in region G. The suppression of TBs increases markedly with a further increase in the loading phase, as shown in Fig. 14b2,b3. For a large TB distance of 30.42 Å, the migration of TBs is not observed at all penetration depths; instead, it is the suppression of TBs and the formation of stacking faults and secondary twins, as shown in zone H. In addition, the amorphous structure increases with the imprinting depth increase in all specimens. The results also show a strong recovery of the FCC structure and twinning in all cases at the unloading stage. Therefore, TB controls the plastic deformation, and the migration of TB is the main mechanism that controls the deformation of the NT-polycrystalline structure.

### Evaluations of elastic recovery ratio

Elastic recovery is one of the important characteristics of material after the end of the nanoimprinting process. To evaluate the elastic recovery ratio of the sample after unloading, some parameters are determined as shown in Supplementary Fig. 3. *a* represents the size of pattern at the top, *b* represents the size of pattern at the bottom, *d* is the depth of the pattern. Indexes 1 and 2 specify the dimension of the sample at an imprinting depth of 30 Å and the unloading stage, respectively. The elastic recovery ratio is defined as follows:

1 $$ \eta _{a}  = \left| {\frac{{a_{1}  - a_{2} }}{{a_{1} }}} \right|,~~~~\eta _{b}  = \left| {\frac{{b_{1}  - b_{2} }}{{b_{1} }}} \right|,~~\eta _{h}  = \left| {\frac{{d_{1}  - d_{2} }}{{d_{1} }}} \right|. $$

**Figure 15:**
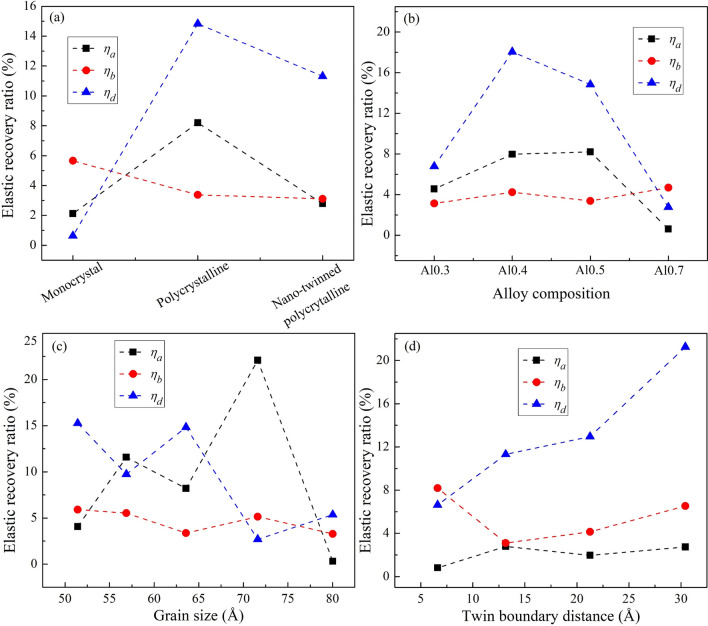
The elastic recovery ratio for various parameters: structures (**a**), alloy compositions (**b**), grain sizes (**c**), TB distances (**d**).

Figure 15a–d displays the elastic recovery ratio for different parameters: crystal structures, alloy compositions, grain sizes, TB distances. The shape of material during the nanoimprinting is dependent on the elastic recovery ratio, and a lower elastic recovery ratio indicates higher material forming ability^64,65^. For different crystal structures, the highest pattern-forming ability is monocrystalline structure because the mean value of elastic recovery ratio is the smallest. The pattern-forming ability of polycrystalline structure is the worst as shown by the elastic recovery ratio much larger than other cases. The highest forming capacity is Al0.7 HEA, and the lowest is Al0.4 HEA with various alloy compositions. For different grain sizes, the result indicates that the elastic recovery ratio is insensitive to change in grain size, in which the smallest elastic recovery ratio is the sample with a grain size 80.03 Å, indicating that the formation ability of pattern is the highest at this grain size. Due to the average elastic recovery ratio is smaller with decreasing the TB distance, so the formability of material is higher with the reduction in TB distance. In general, *η*_*d*_ value is significantly higher than *η*_*a*_ and *η*_*b*_ ratios for all cases, showing that the pattern height has much higher elastic recovery than the width of the pattern.

## Discussion

We have analyzed the influence of the crystal structure and alloy composition on the mechanical properties of FCC AlCoCrFeNi HEA during the nanoimprinting through MD simulations. The results of imprinting force, deformation distribution, structure evolution, dislocation density, atomic displacement, and elastic recovery ratio have clarified the properties of AlCoCrFeNi HEA. The increasing order of maximum imprinting force is polycrystalline, NT-polycrystalline, monocrystalline. This indicates that the presence of GB reduces the strength of the material, while the TB can be improved the material strength. For the alloy composition change, the maximum imprinting force increases as the Al content increases in the alloy. The reverse Hall–Petch relation is found for the polycrystalline structure. Whereas both the Hall–Petch and reverse Hall–Petch relations between the largest imprinting force and the TB distance are shown for the NT-polycrystalline. The plastic deformation reveals that the greater shear strain and the local stress concentrate around the punch. The number of atoms subjected to great shear strain is insensitive to changes in the alloy composition. For polycrystalline and NT-polycrystalline samples, the larger shear strain and stress regions also focus on GB and the contiguous GB area. The specimen with a smaller grain size containing many GB leads to the material softening. Moreover, TB in polycrystalline structure enhances the material stability. The dislocation and stacking fault interact with GB/TB, and boundaries suppress the evolution of dislocation and stacking fault. For polycrystalline structure, the slide and twist of GB play a key role in the deformation mechanism. With the NT-polycrystalline, the TB migration observes during the nanoimprinting. The dislocation density indicates strong growth during the loading stage and a sharp decrease during the unloading phase. The displacement of atoms manifests that the GB suppresses and changes the displacement directions of the atoms; this implies that the GB is the barrier to the movement of atoms. The elastic recovery analysis shows that the formability of pattern is better in monocrystalline than the polycrystalline and NT-polycrystalline. The elastic recovery ratios are not sensitive to changes in alloy composition and grain size with forming ability of Al0.7 HEA and grain size of 80.03 Å higher than that in other cases. Finally, the formability of pattern is higher with reduced TB distance.

## Methods

In the MD models, three atomic groups are established in the sample along the Z direction. Particularly, fixed boundary conditions are imposed on the boundary group, which is composed of the bottom two atomic layers. The thermostat group in the four atomic layers contiguous to the boundary group maintains a specific temperature in the nanoimprinting. The Newtonian group above the thermostat group controls through the canonical ensemble. We first study the influences of various crystal structures, which are monocrystalline (b), polycrystalline (c), and NT-polycrystalline (d) as presented in Fig. 1, on the deformation mechanism of Al_11.0_Co_22.2_Cr_22.2_Fe_22.3_Ni_22.3_ (Al0.5) HEA discovered at a temperature of 300 K. The orientations of the monocrystalline specimen are [1 0 0], [0 1 0] and [0 0 1] along the X-, Y- and Z-direction, respectively. Secondly, the effects of different alloy compositions, which are Al_7.2_Co_23.2_Cr_23.2_Fe_23.2_Ni_23.2_ (Al0.3), Al_9.2_Co_22.7_Cr_22.7_Fe_22.7_Ni_22.7_ (Al0.4), Al_11.0_Co_22.2_Cr_22.2_Fe_22.3_Ni_22.3_ (Al0.5), and Al_11.6_Co_22.3_Cr_22.3_Fe_22.3_Ni_22.3_ (Al0.7), on the mechanical behavior of polycrystalline structure under nanoimprinting are investigated^24^. Thirdly, the influences of grain sizes of the polycrystalline samples containing randomly oriented grains are studied using five different workpieces with the average grain sizes of 80.03; 71.61; 63.57; 56.88; 51.43 Å. Finally, the four models with TB distances are randomly built with the values of 6.61, 13.16, 21.26, and 30.42 Å to evaluate the effect of TB spacing on the mechanistic characteristics for the Al_11.0_Co_22.2_Cr_22.2_Fe_22.3_Ni_22.3_ (Al0.5) HEA.

Before the nanoimprinting process, the specimens are relaxed under the isobaric-isothermal systems (NPT ensemble) in 100 picoseconds (ps) at a temperature of 300 K with a pressure of 0 GPa to reach an initial thermal equilibrium^43,45^. In the nanoimprinting, the punch is moved vertically into the sample at a constant loading speed of 25 m/s along the Z direction to obtain a penetration depth of 30 Å. The periodic boundary conditions apply for the X- and Y-direction, whereas the Z-direction is non-periodic. In addition, the velocity-Verlet-based scheme is chosen to integrate the motion equation with a time step of 1.0 fs. There is an initial space of 10 Å between the sample surface and the punch to avoid errors in the relaxation process due to changes in the volume of the simulation model and the position of the surface atoms.

The embedded atom method (EAM) potential is known as a multibody potential function, and the total potential energy *E*_*p*_ is defined as:

2 $${E}_{p}=\sum_{i=1}^{N}{E}_{i}=\frac{1}{2}{\mathop{\mathop{\sum}\limits_{i,j=1}}\limits_{i\ne j}^{N}}{\varphi }_{ij}\left({r}_{ij}\right)+\sum_{i=1}^{N}{F}_{i}\left({\rho }_{i}\right)$$

where *E*_*i*_ is the potential energy of the atom *i*. The *φ*_*ij*_ represents the potential energy between *i* atoms and *j* atoms, *r*_*ij*_ is the distance between *i* atoms and *j* atoms, and *N* is the total number of atoms in the system. *F*_*i*_ represents the embedding energy required to embed atom *i* into the position where the local electron density is *ρ*_*i*_. The electron density can be calculated as follows:

3 $${\rho }_{i}={\mathop{\mathop{\sum}\limits_{j=1}}\limits_{j\ne i}^{N}}{f}_{j}\left({r}_{ij}\right)$$

here *f*_*i*_(*r*_*ij*_) is the contribution of atom *j* at the site of atom *i* to the electron density.

Therefore, the EAM potential developed by Farkas et al.^66^ is adopted for describing the interatomic interaction between Al–Cr–Co–Fe–Ni in the present study, which is consistent with the interaction of atoms in alloys that have been demonstrated in many previous studies^41,48,67^. The interaction force between the sample and the punch is assisted by Lennard–Jones (LJ) potential^32,64^. The parameters of LJ potential between the punch and the substrate are shown in Table 2. Meanwhile, the atomic interaction of the punch is ignored. The parameters of sample and MD simulation settings is given in Table 1. We have used the simulation platform developed by Plimpton et al.^68^ of Large-scale atomic/molecular massively parallel simulator (LAMMPS) to perform nanoimprinting simulations. The open visualization software (OVITO-version 3.3.5 https://www.ovito.org/about/version-history/) is applied to visualize the mechanical properties in the nanoimprinting as shear strain, local stress, dislocation analysis (DXA), and common neighbor analysis (CNA)^69^.

**Table 1:** The parameters of sample and MD simulation of nanoimprinting.

Parameter	Workpiece	Tool
Material type	Al_x_CoCrFeNi HEA	Diamond
Dimensions (Å)	200 × 55 × 110 (L × W × H)	
Number of atoms	113,088–114,601	57,888
Alloy compositions	Al_7.2_Co_23.2_Cr_23.2_Fe_23.2_Ni_23.2_, Al_9.2_Co_22.7_Cr_22.7_Fe_22.7_Ni_22.7_, Al_11.0_Co_22.2_Cr_22.2_Fe_22.3_Ni_22.3_, Al_11.6_Co_22.1_Cr_22.1_Fe_22.1_Ni_22.1_	
Grain sizes (Å)	80.03; 71.61; 63.57; 56.88; 51.43	
Twin boundary distance (Å)	6.61; 13.16; 21.26; 30.42	
**Parameters of nanoimprinting process**		
Loading speed (m/s)	25	
Indentation depth (Å)	30	
Initial temperature (K)	300

**Table 2:** Lennard–Jones (LJ) potential parameters considered in current study.

Interaction	*ε* (eV)	*σ* (Å)
C–Al	0.108	2.860
C–Co	0.124	2.703
C–Cr	0.123	2.718
C–Fe	0.126	2.711
C–Ni	0.125	2.691

The atomic shear strain and local stress of the substrate are analyzed to investigate the deformation behavior of the samples during nanoimprinting. The von Mises stress is a scalar value that is calculated from normal stresses and shear stresses. The von Mises stress is given by following formula^70,71^:

4 $${\sigma }_{vM}=\sqrt{3\left({\sigma }_{xy}^{2}+{\sigma }_{yz}^{2}+{\sigma }_{zx}^{2}\right)+\frac{1}{2}\left[{\left({\sigma }_{xx}-{\sigma }_{yy}\right)}^{2}+{\left({\sigma }_{xx}-{\sigma }_{zz}\right)}^{2}+{\left({\sigma }_{zz}-{\sigma }_{yy}\right)}^{2}\right]}$$

where *σ*_*xx*_, *σ*_*yy*_, and *σ*_*zz*_ are normal stress in X, Y and Z direction, respectively. *σ*_*xy*_, *σ*_*yz*_, and *σ*_*zx*_ signify shear stress in XY, YZ, and ZX direction, respectively.

Meanwhile, the atomic strain analysis determined through the von Mises shear strain invariant based on previous reports^72,73^ is calculated as follows:

5 $${\eta }_{i}^{Mises}=\sqrt{{\eta }_{xy}^{2}+{\eta }_{yz}^{2}+{\eta }_{zx}^{2}+{\frac{1}{6}[({\eta }_{xx}-{\eta }_{yy})}^{2}+{({\eta }_{yy}-{\eta }_{zz})}^{2}+{({\eta }_{zz}-{\eta }_{xx})}^{2}]}$$

here *η*_*xy*_, *η*_*yz*_, *η*_*zx*_, *η*_*xx*_, *η*_*yy*_, and *η*_*zz*_ are six components of the atomic strain tensor.

## Supplementary information

Supplementary Figures.

## References

[1] Lu K (2010). The future of metals. Science.

[2] Yeh JW (2004). Nanostructured high-entropy alloys with multiple principal elements: novel alloy design concepts and outcomes. Adv. Eng. Mater..

[3] Senkov ON, Miller JD, Miracle DB, Woodward C (2015). Accelerated exploration of multi-principal element alloys with solid solution phases. Nat. Commun..

[4] Zhang Y (2014). Microstructures and properties of high-entropy alloys. Prog. Mater. Sci..

[5] Cantor B, Chang ITH, Knight P, Vincent AJB (2004). Microstructural development in equiatomic multicomponent alloys. Mater. Sci. Eng. A.

[6] Senkov ON, Wilks GB, Scott JM, Miracle DB (2011). Mechanical properties of Nb_25_Mo_25_Ta_25_W_25_ and V_20_Nb_20_Mo_20_Ta_20_W_20_ refractory high entropy alloys. Intermetallics.

[7] Gludovatz B (2014). A fracture-resistant high-entropy alloy for cryogenic applications. Science.

[8] Liu WH (2015). Effects of Nb additions on the microstructure and mechanical property of CoCrFeNi high-entropy alloys. Intermetallics.

[9] Chen MR (2006). Microstructure and properties of Al_0.5_CoCrCuFeNiTix (x= 0–2.0) high-entropy alloys. Mater. Trans..

[10] Zhang K, Fu Z (2012). Effects of annealing treatment on phase composition and microstructure of CoCrFeNiTiAl_x_ high-entropy alloys. Intermetallics.

[11] Tong CJ (2005). Microstructure characterization of Al_x_CoCrCuFeNi high-entropy alloy system with multiprincipal elements. Metall. Mater. Trans. A Phys. Metall. Mater. Sci..

[12] Shun TT, Du YC (2009). Microstructure and tensile behaviors of FCC Al_0.3_CoCrFeNi high entropy alloy. J. Alloys Compd..

[13] Saito T (2003). Multifunctional alloys obtained via a dislocation-free plastic deformation mechanism. Science.

[14] Oses C, Toher C, Curtarolo S (2020). High-entropy ceramics. Nat. Rev. Mater..

[15] Zhao SF, Yang GN, Ding HY, Yao KF (2015). A quinary Ti–Zr–Hf–Be–Cu high entropy bulk metallic glass with a critical size of 12 mm. Intermetallics.

[16] Guo Y, Liu Q (2018). MoFeCrTiWAlNb refractory high-entropy alloy coating fabricated by rectangular-spot laser cladding. Intermetallics.

[17] Hsieh KC (2009). The microstructure and phase equilibrium of new high performance high-entropy alloys. J. Alloys Compd..

[18] Varalakshmi S, Kamaraj M, Murty BS (2010). Processing and properties of nanocrystalline CuNiCoZnAlTi high entropy alloys by mechanical alloying. Mater. Sci. Eng. A.

[19] Chou HP, Chang YS, Chen SK, Yeh JW (2009). Microstructure, thermophysical and electrical properties in Al_x_CoCrFeNi (0≤x≤2) high-entropy alloys. Mater. Sci. Eng. B.

[20] Kao YF, Lee TD, Chen SK, Chang YS (2010). Electrochemical passive properties of Al_x_CoCrFeNi (x= 0, 0.25, 0.50, 1.00) alloys in sulfuric acids. Corros. Sci..

[21] Lin Y (2020). Enhanced radiation tolerance of the Ni-Co-Cr-Fe high-entropy alloy as revealed from primary damage. Acta Mater..

[22] Wang J (2020). Ultrahigh hardness with exceptional thermal stability of a nanocrystalline CoCrFeNiMn high-entropy alloy prepared by inert gas condensation. Scr. Mater..

[23] Kumar N (2015). High strain-rate compressive deformation behavior of the Al_0.1_CrFeCoNi high entropy alloy. Mater. Des..

[24] Wang WR (2012). Effects of Al addition on the microstructure and mechanical property of Al_x_CoCrFeNi high-entropy alloys. Intermetallics.

[25] Ma SG, Zhang SF, Gao MC, Liaw PK, Zhang Y (2013). A successful synthesis of the CoCrFeNiAl_0.3_ single-crystal, high-entropy alloy by Bridgman solidification. Jom.

[26] Li C, Li JC, Zhao M, Jiang Q (2010). Effect of aluminum contents on microstructure and properties of Al_x_CoCrFeNi alloys. J. Alloys Compd..

[27] Shi Y, Collins L, Balke N, Liaw PK, Yang B (2018). In-situ electrochemical-AFM study of localized corrosion of Al_x_CoCrFeNi high-entropy alloys in chloride solution. Appl. Surf. Sci..

[28] Chao Q (2017). Direct laser deposition cladding of Al_x_CoCrFeNi high entropy alloys on a high-temperature stainless steel. Surf. Coat. Technol..

[29] Doan DQ, Fang TH, Chen TH (2021). Machining mechanism and deformation behavior of high-entropy alloy under elliptical vibration cutting. Intermetallics.

[30] Doan DQ, Fang TH, Chen TH (2020). Nanotribological characteristics and strain hardening of amorphous Cu_64_Zr_36_/crystalline Cu nanolaminates. Tribol. Int..

[31] Doan DQ, Fang TH, Tran AS, Chen TH (2020). High deformation capacity and dynamic shear band propagation of imprinted amorphous Cu_50_Zr_50_/crystalline Cu multilayered nanofilms. J. Phys. Chem. Solids.

[32] Xie L, Brault P, Thomann AL, Bauchire JM (2013). AlCoCrCuFeNi high entropy alloy cluster growth and annealing on silicon: A classical molecular dynamics simulation study. Appl. Surf. Sci..

[33] Alhafez IA, Ruestes CJ, Bringa EM, Urbassek HM (2019). Nanoindentation into a high-entropy alloy—an atomistic study. J. Alloys Compd..

[34] Chou SY, Krauss PR, Renstrom PJ (1995). Imprint of sub-25 nm vias and trenches in polymers. Appl. Phys. Lett..

[35] Chou SY, Krauss PR, Renstrom PJ (1996). Imprint lithography with 25-nanometer resolution. Science.

[36] Sun X, Zhuang L, Zhang W, Chou SY (1998). Multilayer resist methods for nanoimprint lithography on nonflat surfaces. J. Vac. Sci. Technol. B.

[37] Schulz H (2000). New polymer materials for nanoimprinting. J. Vac. Sci. Technol. B.

[38] Liu X (2015). Die imprinting of MGs: a one-step approach for large-area metallic photonic crystals. Mater. Des..

[39] Hirel P (2015). Atomsk: A tool for manipulating and converting atomic data files. Comput. Phys. Commun..

[40] Brostow W, Dussault JP, Fox BL (1978). Construction of Voronoi polyhedra. J. Comput. Phys..

[41] Fourmont A, Le Gallet S, Politano O, Desgranges C, Baras F (2020). Effects of planetary ball milling on AlCoCrFeNi high entropy alloys prepared by Spark Plasma Sintering: Experiments and molecular dynamics study. J. Alloys Compd..

[42] Butler TM, Weaver ML (2016). Oxidation behavior of arc melted AlCoCrFeNi multi-component high-entropy alloys. J. Alloys Compd..

[43] Wu CD, Hou CJ (2018). Molecular dynamics analysis of plastic deformation and mechanics of imprinted metallic glass films. Comput. Mater. Sci..

[44] Fang TH, Wu CD, Chang WJ (2007). Molecular dynamics analysis of nanoimprinted Cu–Ni alloys. Appl. Surf. Sci..

[45] Doan DQ, Fang TH, Chen TH (2020). Effects of grain and twin boundary on friction and contact characteristics of CuZrAl nanocrystallines. Appl. Surf. Sci..

[46] Li J (2016). Study of nanoindentation mechanical response of nanocrystalline structures using molecular dynamics simulations. Appl. Surf. Sci..

[47] Huang CC, Chiang TC, Fang TH (2015). Grain size effect on indentation of nanocrystalline copper. Appl. Surf. Sci..

[48] Doan DQ, Fang TH, Chen TH (2020). Influences of grain size and temperature on tribological characteristics of CuAlNi alloys under nanoindentation and nanoscratch. Int. J. Mech. Sci..

[49] Rojas-Nunez J (2020). Polycrystalline Ni nanotubes under compression: a molecular dynamics study. Sci. Rep..

[50] Li J, Fang Q, Liu B, Liu Y (2016). The effects of pore and second-phase particle on the mechanical properties of machining copper matrix from molecular dynamic simulation. Appl. Surf. Sci..

[51] Li J, Fang Q, Liu B, Liu Y, Liu Y (2016). Mechanical behaviors of AlCrFeCuNi high-entropy alloys under uniaxial tension via molecular dynamics simulation. RSC Adv..

[52] Tang Y, Bringa EM, Meyers MA (2013). Inverse Hall-Petch relationship in nanocrystalline tantalum. Mater. Sci. Eng. A.

[53] Lv Y (2017). Cooling rate effect on microstructure and mechanical properties of Al_x_CoCrFeNi high entropy alloys. Mater. Des..

[54] Liu Y, Ngan AW (2001). Depth dependence of hardness in copper single crystals measured by nanoindentation. Scr. Mater..

[55] Pan Z, Li Y, Wei Q (2008). Tensile properties of nanocrystalline tantalum from molecular dynamics simulations. Acta Mater..

[56] Chen S (2020). Hall–Petch and inverse Hall–Petch relations in high-entropy CoNiFeAl_x_Cu_1-x_ alloys. Mater. Sci. Eng. A.

[57] Zhang L, Shibuta Y (2020). Inverse Hall–Petch relationship of high-entropy alloy by atomistic simulation. Mater. Lett..

[58] Jung BB, Lee HK, Park HC (2013). Effect of grain size on the indentation hardness for polycrystalline materials by the modified strain gradient theory. Int. J. Solids Struct..

[59] Tian Y, Fang Q, Li J (2020). Molecular dynamics simulations for nanoindentation response of nanotwinned FeNiCrCoCu high entropy alloy. Nanotechnology.

[60] Qi Y (2021). Effects of microstructure and temperature on the mechanical properties of nanocrystalline CoCrFeMnNi high entropy alloy under nanoscratching using molecular dynamics simulation. J. Alloys Compd..

[61] Pande CS, Cooper KP (2009). Nanomechanics of Hall-Petch relationship in nanocrystalline materials. Prog. Mater. Sci..

[62] Afanasyev KA, Sansoz F (2007). Strengthening in gold nanopillars with nanoscale twins. Nano Lett..

[63] Tian Y (2020). Plastic deformation mechanisms of tension-compression asymmetry of nano-polycrystalline tial: Twin boundary spacing and temperature effect. Comput. Mater. Sci..

[64] Doan DQ, Fang TH, Tran AS, Chen TH (2019). Residual stress and elastic recovery of imprinted Cu-Zr metallic glass films using molecular dynamic simulation. Comput. Mater. Sci..

[65] Wu CD, Fang TH, Chen CY, Weng CI (2014). Effect of nanograin size on nanoformed NiTi alloys. Appl. Surf. Sci..

[66] Farkas D, Caro A (2020). Model interatomic potentials for Fe–Ni–Cr–Co–Al high-entropy alloys. J. Mater. Res..

[67] Bahramyan M, Mousavian RT, Brabazon D (2020). Determination of atomic-scale structure and compressive behavior of solidified Al_x_CrCoFeCuNi high entropy alloys. Int. J. Mech. Sci..

[68] Plimpton S (1995). Fast parallel algorithms for short-range molecular dynamics. J. Comput. Phys..

[69] Stukowski A (2009). Visualization and analysis of atomistic simulation data with OVITO–the Open Visualization Tool. Model. Simul. Mater. Sci. Eng..

[70] Toyohara R (2020). Finite element analysis of load transition on sacroiliac joint during bipedal walking. Sci. Rep..

[71] Goel S, Beake B, Chan CW, Faisal NH, Dunne N (2015). Twinning anisotropy of tantalum during nanoindentation. Mater. Sci. Eng. A.

[72] Shimizu, F., Ogata, S. & Li, J. Theory of shear banding in metallic glasses and molecular dynamics calculations. *Mater. Trans.* **48**, 2923–2927 (2007).

[73] Arman B, Luo SN, Germann TC, Çağın T (2010). Dynamic response of Cu 46 Zr 54 metallic glass to high-strain-rate shock loading: Plasticity, spall, and atomic-level structures. Phys. Rev. B.

