# Supplementary material for: Microstructure and composition dependence of mechanical characteristics of nanoimprinted AlCoCrFeNi high-entropy alloys
Source: Sci Rep. 2021 Jul 1;11:13680. doi: 10.1038/s41598-021-93272-y (PMC8249401; doi:10.1038/s41598-021-93272-y)
Supplement: Supplementary file 1 — Supplementary Figures. [file 41598_2021_93272_MOESM1_ESM.docx]

Supplementary Information for

**Microstructure and composition dependence of mechanical characteristics of nanoimprinted AlCoCrFeNi high-entropy alloys**

Dinh-Quan Doan^1,2^, Te-Hua Fang^1,*^, Tao-Hsing Chen^1^

^1^Department of Mechanical Engineering, National Kaohsiung University of Science and Technology, Kaohsiung 807, Taiwan

^2^Faculty of Mechanical Engineering, Hung Yen University of Technology and Education, Khoai Chau District, Hung Yen Province, Vietnam

* Te-Hua Fang; [fang.tehua@msa.hinet.net](mailto:fang.tehua@msa.hinet.net)


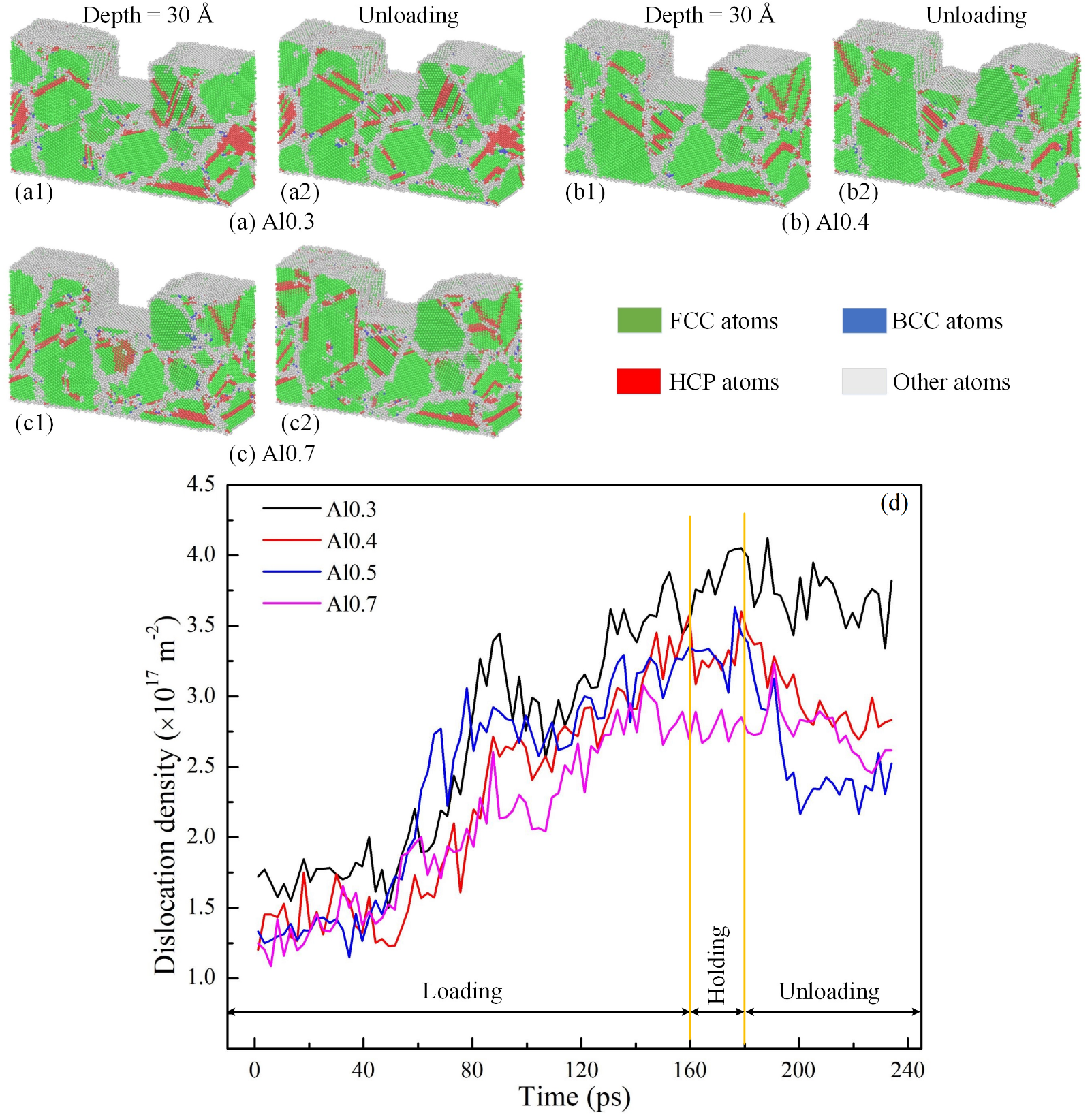


**Supplementary Fig. 1.** The microstructure snapshots at an imprinting depth of 30 Å and the unloading stage for the polycrystalline structure of the Al_x_CoCrFeNi HEA with different alloy compositions: Al0.3 (a); Al0.4 (b); and Al0.7 (c). Development of the total dislocation density of the Al_x_CoCrFeNi HEA during the nanoimprinting with different alloy compositions (d).


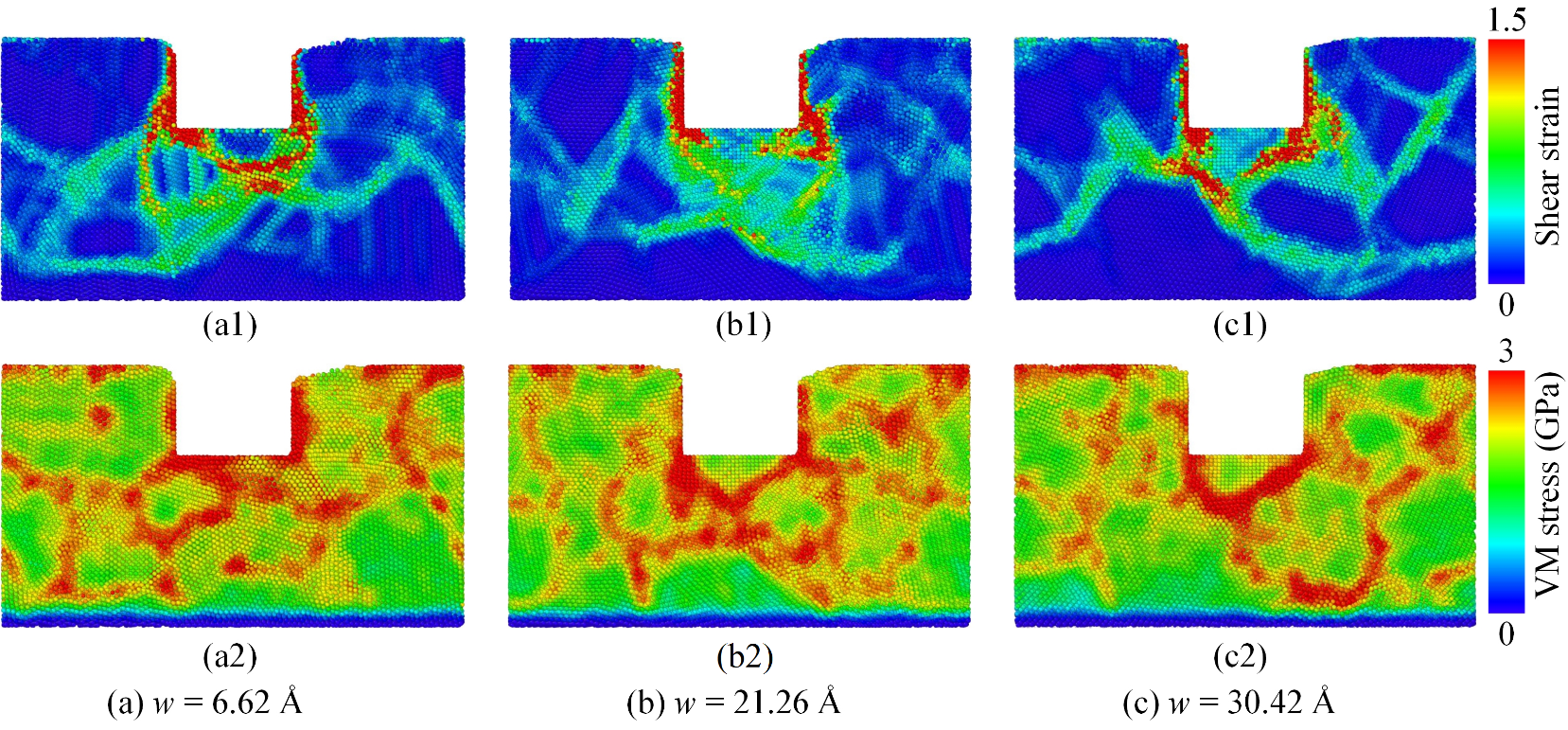


**Supplementary Fig. 2.** The cross-section of atomic shear strain distributions (a1-c1) and the local stress distributions of atoms (a2-c2) for NT-polycrystalline structure of the Al0.5 HEA at an imprinting depth of 30 Å with various TB distances: 6.61 Å (a); 21.26 Å(b); and 30.42 Å (c).


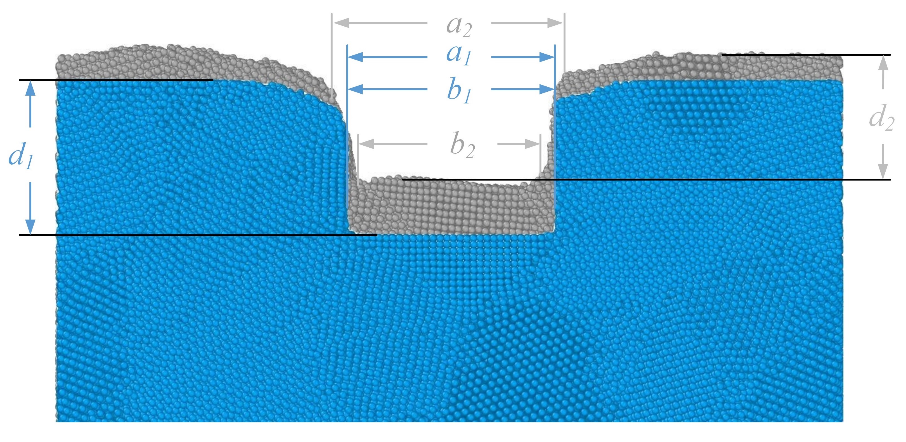


**Supplementary Fig. 3.** Definition of the sample parameters before and after elastic recovery.
